# HIV-genetic diversity and drug resistance transmission clusters in Gondar, Northern Ethiopia, 2003-2013

**DOI:** 10.1371/journal.pone.0205446

**Published:** 2018-10-10

**Authors:** Dawit Assefa Arimide, Almaz Abebe, Yenew Kebede, Fekadu Adugna, Tesfaye Tilahun, Desta Kassa, Yibeltal Assefa, Taye Tolera Balcha, Per Björkman, Patrik Medstrand

**Affiliations:** 1 Department of Translational Medicine, Lund University, Malmö, Sweden; 2 The Ethiopian Public Health Institute, Addis Ababa, Ethiopia; 3 Center for Disease Control and Prevention (CDC)-Ethiopia, Addis Ababa, Ethiopia; 4 World Health Organization (WHO)-Ethiopia, Addis Ababa, Ethiopia; 5 School of Public Health, University of Queensland, Brisbane, Australia; 6 Arman Hansen research Institute (AHRI), Addis Ababa, Ethiopia; University of Cincinnati College of Medicine, UNITED STATES

## Abstract

**Background:**

The HIV-1 epidemic in Ethiopia has been shown to be dominated by two phylogenetically distinct subtype C clades, the Ethiopian (C’-ET) and East African (C-EA) clades, however, little is known about the temporal dynamics of the HIV epidemic with respect to subtypes and distinct clades. Moreover, there is only limited information concerning transmission of HIV-1 drug resistance (TDR) in the country.

**Methods:**

A cross-sectional survey was conducted among young antiretroviral therapy (ART)-naïve individuals recently diagnosed with HIV infection, in Gondar, Ethiopia, 2011–2013 using the WHO recommended threshold survey. A total of 84 study participants with a median age of 22 years were enrolled. HIV-1 genotyping was performed and investigated for drug resistance in 67 individuals. Phylogenetic analyses were performed on all available HIV sequences obtained from Gondar (n = 301) which were used to define subtype C clades, temporal trends and local transmission clusters. Dating of transmission clusters was performed using BEAST.

**Result:**

Four of 67 individuals (6.0%) carried a HIV drug resistance mutation strain, all associated with non-nucleoside reverse transcriptase inhibitors (NNRTI). Strains of the C-EA clade were most prevalent as we found no evidence of temporal changes during this time period. However, strains of the C-SA clade, prevalent in Southern Africa, have been introduced in Ethiopia, and became more abundant during the study period. The oldest Gondar transmission clusters dated back to 1980 (C-EA), 1983 (C-SA) and 1990 (C’-ET) indicating the presence of strains of different subtype C clades at about the same time point in Gondar. Moreover, some of the larger clusters dated back to the 1980s but transmissions within clusters have been ongoing up till end of the study period. Besides being associated with more sequences and larger clusters, the C-EA clade sequences were also associated with clustering of HIVDR sequences. One cluster was associated with the G190A mutation and showed onward transmissions at high rate.

**Conclusion:**

TDR was detected in 6.0% of the sequenced samples and confirmed pervious reports that the two subtype C clades, C-EA and C’-ET, are common in Ethiopia. Moreover, the findings indicated an increased diversity in the epidemic as well as differences in transmission clusters sizes of the different clades and association with resistance mutations. These findings provide epidemiological insights not directly available using standard surveillance and may inform the adjustment of public health strategies in HIV prevention in Ethiopia.

## Introduction

The global scale up of antiretroviral therapy (ART) has resulted in decline in HIV related morbidity, mortality and HIV transmission. In most low and middle income countries (LMIC) standardized first line antiretroviral regimens are used, consisting of two nucleoside reverse transcriptase inhibitor (NRTI) and one non-nucleoside reverse transcriptase inhibitor (NNRTI)] [1–3]. The emergence of HIV drug resistance (HIVDR), particularly towards drugs with low genetic barriers, eg. NNRTIs, has been shown to increase with time after introduction of ART programs [4]. One explanation for this is likely to be the lack of routine monitoring of plasma viral load, which has not been scaled up at the same rate as ART service expansion (ART roll-out) [2–9]. This leads to delayed identification of patients with treatment failure, with risk of accumulation of drug resistance mutations (DRM) in such individuals. Furthermore, individuals with unrecognized virological treatment failure are potential reservoirs for onwards transmission of viruses with DRM (commonly referred to as transmitted drug resistance; TDR) [2, 9–11]. If this occurs in populations with high incidence and at high risk of onward transmissions, the prevalence of drug resistant HIV strains may increase and be further amplified in the population [12]. Thus, the emergence of drug resistant viral strains constitutes a threat to the outcome of ART programs [13, 14]. Virological monitoring and resistance surveillance is therefore a priority [2].

The first HIV positive sera and AIDS case was diagnosed in Ethiopia in 1984 and 1986, respectively [15, 16]. By the late 1980s a high prevalence of HIV-1 was detected among commercial sex workers and among long distance truck drivers [17]. ART was introduced in the public health sector in 2003, with free ART provision since 2005 [18]. In 2017, an estimated ~740,000 individuals were living with HIV and ~426,000 had initiated ART [19]. With the scale up and decentralization of the service in Ethiopia, the emergence and transmission of resistance is expected, as has been evident in other LMIC [4–7]. Following the WHO recommendation, Ethiopia implemented a strategy for the prevention and monitoring of HIVDR to maximize the durable efficacy of affordable and potent first-line ART regimens [20].

A threshold survey was performed according to WHO guideline in the capital Addis Ababa, the city where ART was first started in Ethiopia, among treatment naïve women attending antenatal clinics in 2005. This survey revealed no major drug resistance for any available HIV drug class [21]. A nationwide study 2009–2011 indicated a pretreatment DR (PDR) level of 3.9%[22]. Moreover, three studies evaluated PDR among patients in Gondar (located in Northern Ethiopia) [23–25]., Even though these studies were not performed according to the WHO threshold surveillance method, they indicated an increase of PDR after ART roll out in the country.

The Ethiopian HIV epidemic is dominated by HIV subtype C, similar to the countries of Southern Africa, and although Ethiopia shares borders with countries were subtypes A and D are common, these subtypes have rarely been identified in Ethiopia [26]. Previous studies have shown that two distinct subtype C strains are co-circulating in Ethiopia, designated C and C’ [27], and recombinant forms of the C and C’ strains [28]. Ten distinct subtype C clades (termed C1-C10) have been defined based on phylogenetic relationship. Clades C1-C9 were mainly represented by sequences obtained from countries in southern Africa while the C10 clade represented sequences from East and Central Africa[29]. Further molecular characterization revealed that the Ethiopian C strain were similar to strains circulating in other East African countries while the C’ strain were shown to represented a distinct subclade of a southern African clade. Thus, based on previous phylogenetic studies, the African subtype C stains can be divided into three major groups: the southern African subtype C clades (C-SA), the Ethiopian C’ clade (C’-ET), and the central and east African subtype C clade (C-EA) [29, 30]. Previous phylodynamic studies have indicated that HIV-1 was introduced in Ethiopia in the late 1960s to the early 1970s, more than 10 years before the first documented AIDS case [30, 31].

The aim of this study was to estimate the prevalence of TDR in young adults with assumed recent HIV-1 infection using the WHO threshold surveillance method, to describe the molecular epidemiology of HIV-1 in terms of genetic diversity, transmission clusters and DRM transmissions within clusters in Gondar, Ethiopia.

## Methods

### Study design and site selection

A cross-sectional survey was conducted between August 2011 and December 2013 among antiretroviral-naive adults to evaluate transmitted drug resistance, according to the World Health Organization (WHO)-recommended threshold survey methodology [13]. It was conducted in two of the major Voluntary Counselling and Testing (VCT) clinics in Gondar, located 700 km north of Addis Ababa. Gondar is the second largest town in Amhara region and, it was one of the areas in the country where public ART was first initiated (in year 2003). The HIV-prevalence 2003–2013 in Gondar was on a stable level (mean: 10.6%) while the national prevalence during the same time period showed a declining trend, from 12% to 4.4% [32]. Furthermore, three studies conducted at the hospital in 2003–2010 showed the presence of HIVDR in the area [23–25]. The sample size for the current survey followed the sequential sampling method selected by WHO for the surveillance of transmitted HIVDR in low-resource settings. According to the recommendation, it is advised to collect about 70 specimens of eligible individuals consecutively diagnosed with HIV in sites within a survey area [20].

### Study participants

Individuals with new diagnosed HIV infection, among VCT clients at the two survey sites were asked to participate in the study. By adopting the WHO recommended inclusion criterion, participants 18–25 years old, who had no prior history of HIV/AIDS-related illness and no history of ART, resident of Gondar for more than a year, and had no history of previous pregnancy were consecutively enrolled. After obtaining written informed consent, 10 ml of blood was collected by venepuncture. Screening for HIV was done using point of care rapid testing format employed for HIV diagnosis in Ethiopia. This algorithm uses HIV (1 + 2) Antibody Colloidal Gold (KHB, Shanghai Kehua Bio-engineering Co Ltd, China) as a screening test, followed by HIV 1/2 STAT-PAK (Chembio Diagnostics, USA) if positive. In cases with negative STAT-PAK results following a positive KHB test, a third test, Unigold HIV (Trinity Biotech, Ireland), was used as confirmation. Specimens were transported to Ethiopia Public Health Institute, National HIV laboratory, Addis Ababa (a WHO accredited laboratory for genotyping) on dry ice for long term storage at -80°C until genotyping. At the time of blood sampling basic demographic and clinical information, including age, gender, and history of HIV test was collected using a standardized questionnaire.

### HIV-1 genotyping

A 1084-bp fragment of HIV-1 *pol* (corresponding to the position: 2243–3326 of HXB2, Genbank Accession Number: K03455) comprising amino acids 6–99 of the protease (PR) and 1–251 of the reverse transcriptase (RT) was amplified using an in-house genotyping assay as described in [33], and S1 Text. PCR products were directly sequenced using six primers (three on each strand) on an ABI 3100 or an ABI 3500xl DNA Genetic Analyzer (Applied Biosystems). Sequence assembly and editing were performed using RECall V 2.0 HIV-1 sequencing analysis tool [34].

### Identification of drug resistance mutations

Surveillance drug resistance mutations (SDRMs) were examined according to the Stanford Genotypic Resistance calibrated population resistance (CPR) tool version 6.0 based on the WHO surveillance transmitted drug resistance mutation list of 2009 [35, 36]. Classification of TDR level (low: < 5%, moderate: 5–15%, or high: >15%) was made based on the WHO threshold survey protocol [20].

### HIV-1 subtyping and recombination analysis

Sequence quality control was performed using the online Quality Control program of the Los Alamos HIV sequence database (hiv.lanl.gov). The REGA and Comet online subtyping tools were used for initial classification into subtypes and inter-subtype recombinants [37, 38]. Putative intra-subtype recombinants were verified using Simplot ver. 3.5.1[39]. All sequences were also screened for recombination using RDP ver. 3.44 [40]. Final subtyping was performed through phylogenetic analysis using the reference HIV-1 data set from Los Alamos HIV sequence database (hiv.lanl.gov). Sequences were aligned using ClustalX2 [41] and then edited to a final length of 1044 bases using BioEdit v4.0.6 (http://www.mbio.ncsu.edu/bioedit/bioedit.html). A Maximum likelihood (ML) phylogenetic tree was constructed using the online version of PhyML with the GTR+I+Γ nucleotide substitution model (using estimated proportion of invariable sites and four gamma categories) and NNI plus SPR to estimate the tree topology. Branch support was determined with aLRT-SH (approximate likelihood ratio test Shimodaira-Hasegawa like) implemented in PhyML. A branch in the phylogeny with an aLRT-SH value ≥0.9 was considered significant [42, 43].

To further dissect the subtype C distribution in Gondar, sub-subtyping into subtype C clades and detection of putative intra-subtype C recombinants were performed using sequences obtained from this study population and all previously reported HIV-1 *pol* sequences from Gondar [23–25]. Briefly, reference sequences of the three subtype C clades were obtained as described in S1 Text. Putative intra-subtype recombinant subtype C sequences were identified by jpHMM [44] using parameters as outlined in S1 Text. The final subtyping ML tree is shown in S1 Fig.

### Clade distribution and transmission cluster analysis

For the purpose of a comprehensive analysis of temporal changes of subtype C clades over time in Gondar, subtype C clades were identified using ML phylogenetic tree analysis. For the purpose of transmission cluster analysis, we followed a methodology described previously and included a data set of similar sequences from GenBank by identifying the ten best scoring GenBank sequences with BLAST for each of the Gondar sequences in the study [43, 45, 46]. The final data set contained 491 sequences (n = 299 from Gondar and n = 192 GenBank reference sequences; see S2 Table) which were used to define local (Gondar) transmission clusters. Transmission clusters were defined as described previously [43, 45–48]. Briefly, a transmission cluster was defined as a cluster in the ML phylogeny from root to tips. A cluster with an aLRT SH-support of ≥0.9 that had a majority (at least 80%) of sequences from Gondar was considered as a Gondar (local) transmission cluster. Transmission clusters were defined based on their sizes (number of sequences/cluster), into dyads (two sequences), medium sized clusters/networks (3–14 sequences) and large clusters (≥15 sequences) [43, 49]. Clusters sharing >33% of the same DRM were defined as a putative drug resistance transmission clusters [50].

### Evolutionary and phylodynamic analysis

To estimate an evolutionary rate for each of the three clades which were needed to perform dated cluster analysis using phylodynamic analyses, three data sets were assembled, containing both global and Ethiopian sequences which were considered to be representative for the different clades. This approach has been used previously for HIV-1 subtype B [51, 52]. The analysis was performed using the sequences shown in S1 Fig and S2 Table, by randomly selecting a subset of sequences from each clade (N = 86, N = 65 and N = 70 for C-EA, C’-ET and C-SA, respectively). A maximum of one sequence of each transmission cluster was allowed in the final data set. For the data set containing the southern African clade (C-SA) sequences, the temporal signal was weak (R^2^ = 0.03), as assessed by root-to-tip analysis using TempEst, indicating that the data set was not optimal for estimating reliable substitution rates, while the R^2^ was higher for the C-EA and C’-ET clades (R^2^ = 0.35 and 0.22, respectively), indicating a better temporal signal in the datasets [53]. We therefore estimated the substitution rates for the C-EA and C’-ET sequences only and relied on previous estimates for the C-SA clades (see below). For these analyses we centered the prior mean rate at 0.001 substitutions/site/year and specified the standard deviation to 0.33 on a lognormal distribution such that the 2.5 and 97.5 percentiles of the distribution contained the rates obtained previously of HIV-1 *pol* of subtype C [54, 55]. The evolutionary rate was estimated by employing the Bayesian Markov Chains Monte Carlo (MCMC) clock method implemented in the BEAST software package v1.8.4 [56]. We estimated the evolutionary rate using both a strict and relaxed Bayesian MCMC clock, in both cases with a flexible demographic model (the Bayesian skyline plot) as a tree prior. This analysis estimated posterior distribution of median rates for the C-EA and C’-ET clades to 1.26 x 10^−3^ (95% HPD: 8.80x10^-4^–1.64x10^-3^) and 1.44x10^-3^ (95% HPD: 9.70x10^-4^–1.91x10^-3^) substitutions/site/year, respectively (S1 Table). Using these rates as priors, subsequent BEAST analysis was performed on the C-EA and C’-ET sequences obtained from Gondar only while a previous evolutionary rate of 2.15x10-3 (1.79x10^-3^–2.60x10^-3^, 95% HPD) substitutions/site/year was used as a prior for the C-SA sequences of Gondar [54]. In all cases, a lognormal distribution on the rate prior was employed, as described above, such that the prior mean rate was centered at each clade-specific mean rate and that the 2.5 and 97.5 percentiles of the distribution contained the rates obtained from the above analyses (S1 Table). For all analyses we used both strict and relaxed clock models and specified a codon position partitioning model (the SRD06 model) for all data sets [57]. We also employed different demographic tree priors on each data set, the skyride, the logistic and exponential demographic models implemented in BEAST (S1 Table). Model comparisons were done by Bayes factor (BF) analysis of marginal likelihoods [58]. BF values larger than 3 and 5 were considered to represent strong and very strong evidence respectively, against H_0_ [59].

### Statistical analysis

Statistical tests were performed using SPSS 24 (IBM Corp., Armonk, NY, USA). DR prevalence was determined with a confidence interval (CI) of 95% using the Wilson method. Categorical variables were compared using 2-tailed Fisher’s exact test, while continuous variables were compared using Mann-Whitney 2-tailed U test. Trends over time were analysed using linear-by-linear test for association.

### Ethical approval

Scientific and ethical approval was granted by the Research and Ethical Clearance Committee of the Ethiopian Public Health Institute, and the National Health Research Ethics Review Committee of Ministry of Science and Technology of Ethiopia. All participants provided written informed consent.

### Availability of data

Nucleotide sequences reported in this study have been deposited in the Genbank repository (Accession Numbers: KM390990—KM391398).

## Results

### Population characteristics

A total of 84 individuals attending the VCT clinics at Gondar University hospital (n = 64) and Gondar health centre (n = 20) met the inclusion criteria and were enrolled in the study.

All samples were confirmed as HIV-1 positive. In total, 78% (67/84) of the samples were successfully amplified and sequenced. The median age among the 84 individuals was 22 years (IQR: 20–23) and 83% were females, which was similar to the age (22 years [IQR: 20–24]) and gender distribution (87% females) of the 67 participants whose samples were successfully genotyped (p = 0.992 and p = 0.653, M-W and Fisher exact test, respectively).

### Levels of transmitted drug resistance

TDR levels were determined from the 67 sequenced samples. Four specimens (6.0%) carried major DRMs, corresponding to a moderate TDR level [60]. Two had K103N, one G190S and one Y181C, all representing resistance to non-nucleoside reverse transcriptase inhibitors (NNRTI). No NRTI or protease inhibitor (PI) associated DRMs were identified among the sequenced samples (Table 1). Although there were more females enrolled in the survey than men there were no association between gender and DRMs (2 men and 2 females carried HIVDR virus; p = 0.084, FET).

**Table 1:** Prevalence of HIV drug resistance mutations in Gondar 2003–2013.

Drug class and DRM^1^	Populations^2^			
	Before ART, 2003 (n = 92)	General, 2009 (n = 158)	General, 2010 (n = 61)	Young (present study), 2011–2013 (n = 67)
**All classes**	3 (**3.3**, 1.1–9.2)	8 (**5.1**, 2.6–9.7)	5 (**8.2**, 3.6–17.8)	4 (**6.0**, 2.4–14.4)
**PI**	1 (1.0, 0.-5.9)	2 (1.3, 0.4–4.5)	1 (1.6, 0.3–8.7)	0 (0.0, 0.0–5.4)
M46I	-	1	1	-
F53L	-	1	-	-
I85V	1	-	-	-
**NRTI**	0 (0.0, 0.0–4.0)	3 (1.9, 0.7–5.4)	1 (1.6, 0.3–8.7)	0 (0.0, 0.0–5.4)
D67E	-	1	-	-
M184I	-	-	1	-
L210W	-	2	-	-
**NNRTI**	2 (2.2, 0.6–7.6)	3 (1.9, 0.7–5.4)	3 (4.9, 1.7–13.5)	4 (6.0, 2.4–14.4)
K101E	-	1	-	-
K103N	-	-	-	2
Y181I	-	-	1	-
Y181C	-	-	-	1
G190A	2	2	1	-
G190E	-	-	1	-
G190S	-	-	-	1

1: Drug class: PI: protease inhibitors; NRTI: nucleoside reverse transcriptase inhibitor; NNRTI: non-nucleoside reverse transcriptase inhibitors; DRM: drug resistance mutation. Mutations were defined by the Stanford Genotypic Resistance Interpretation Algorithm (http://hivdb.stanford.edu/pages/algs/HIVdb.html) using the calibrated population resistance (CPR) tool version 6.0 (http://cpr.stanford.edu/cpr/servlet/CPR), based on the WHO surveillance transmitted drug resistance mutation list of 2009.

2: Study Population, year of sample collection (number of samples genotyped). Number of drug resistance mutations for all or per class (**% DRM**, 95% CI) or number of specific drug resistance mutations. Nucleotide sequences were obtained from this and previous studies in Gondar [23–25]; 2003, Kassu et al. (2007); 2009, Mulu et al. (2009); 2010, Huruy et al. (2015); 2011–2013, this study.

### Level of HIV-1 drug resistance in Gondar 2003–2013

Analysis of HIV DRMs of two previous studies from Gondar after ART roll out in 2009 and 2010 as described in Methods, revealed levels comparable to our threshold study (4.4–8.2% versus 6.0%; Table 1) [23, 25]. These estimates were higher than the levels reported in the study performed before ART roll-out in Gondar 2003 (3.3%) [24]. However, the confidence intervals of the levels of DRMs were largely overlapping between the studies and the proportion of DRMs before and after ART roll out were not different (p = 0.584, FET; Table 1). DRMs of the previous three studies were as in the present study, most often associated with NNRTIs. Taken together, the G190A/S/E mutations were most prominent (seven of the 12 NNRTI mutations), followed by the K103N, Y181I/C and K101E substitutions (two, two and one, respectively). Mutations conferring resistance to NRTIs and PIs were found in earlier studies at low levels (1.0–1.9%) but were not identified in the present study (Table 1).

### Subtype C clade distribution and temporal changes 2003–2013

**Fig 1:**
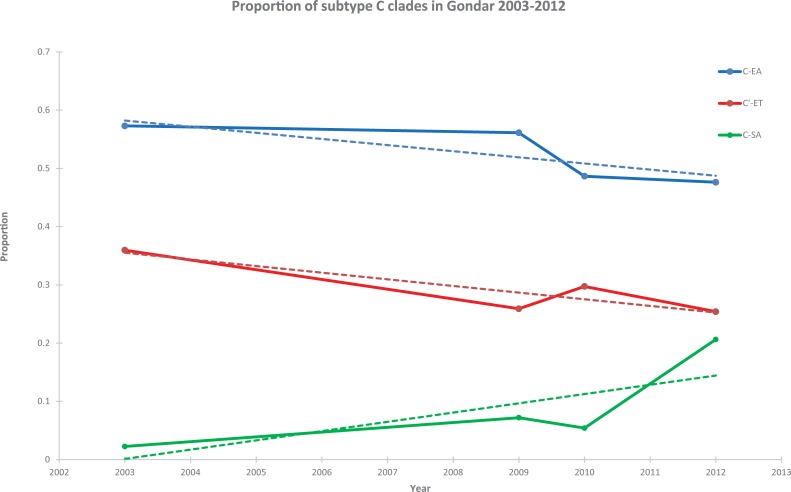
Trend analysis of subtype C clades 2003–2013. Proportion of different subtype C clades in Gondar 2003–2013. A dotted line indicates the trend over time. The 2012 time point included specimens obtained 2011–2013 (current study).

Phylogenetic subtyping revealed that 94% (63 of 67) of study participants enrolled 2011–2013 were infected with HIV-1 subtype C, while the remaining four study participants had subtype A, B or A/C recombinant sequences (S2 Table). Since previous observations have suggested more rapid expansion of the C’-ET strains than the C-EA strains in Ethiopia, we further investigated the distribution of our sequences into different subtype C clades [61]. Detailed analysis indicated that 59 of 63 sequences represented the three major subtype C clades: C-EA (n = 30), C’-ET (n = 16) and C-SA (n = 13), while four sequences were putative C-EA/C’-ET recombinants (S2 Table). For in-depth phylogenetic analysis, we next constructed a new data set that represented non-recombinant subtype C sequences of the current study (n = 59), the three previous studies from Gondar in 2003–2010 (n = 242) and a Genbank reference data set (n = 190), resulting in a final data set of 491 sequences collected 1986–2013 (n = 301 from Gondar; S2 Table). Phylogenetic subtyping using all 491 sequences revealed that sequences fell into one of the three subtype C clades (S1 Fig). Among the 301 sequences from Gondar, 59% (n = 177) were classified as C-EA, 32% (n = 97) as C’-ET and 9% (n = 27) as C-SA. Notably, sequences of the C-SA clade have not been described in Ethiopia previously and the proportion of this clade in Gondar increased from 2% in 2003 to 23% in 2012 (p<0.001, two-tailed linear-by-linear test for association) while there was a modest decline of the two major clades (C-EA: 60%-51%; C’-ET: 38%-26%; Fig 1) during the same time period. However, the proportion of C-EA remained higher than C’-ET and C-SA at each time point 2003–2013. Further analysis of the temporal changes revealed that there was no changes of the proportions of the C-EA and C’-ET clades in Gondar during the same time period (p = 0.146 and p = 0.173, respectively, two-tailed linear-by-linear test for association). Among the sequences with DRMs identified in Gondar (Table 1 and S2 Table), 13 were classified as belonging to the C-EA clade, two to the each of the C’-ET and C-SA clades, however, there were no association of DRMs to either of the clades (C-EA vs C’-ET, p = 0.095; C-EA vs C-SA, p = 1.000; and C’-ET vs C-SA, p = 0.210; FET for all comparisons).

### Transmission cluster analysis and association with drug resistance mutations

**Fig 2:**
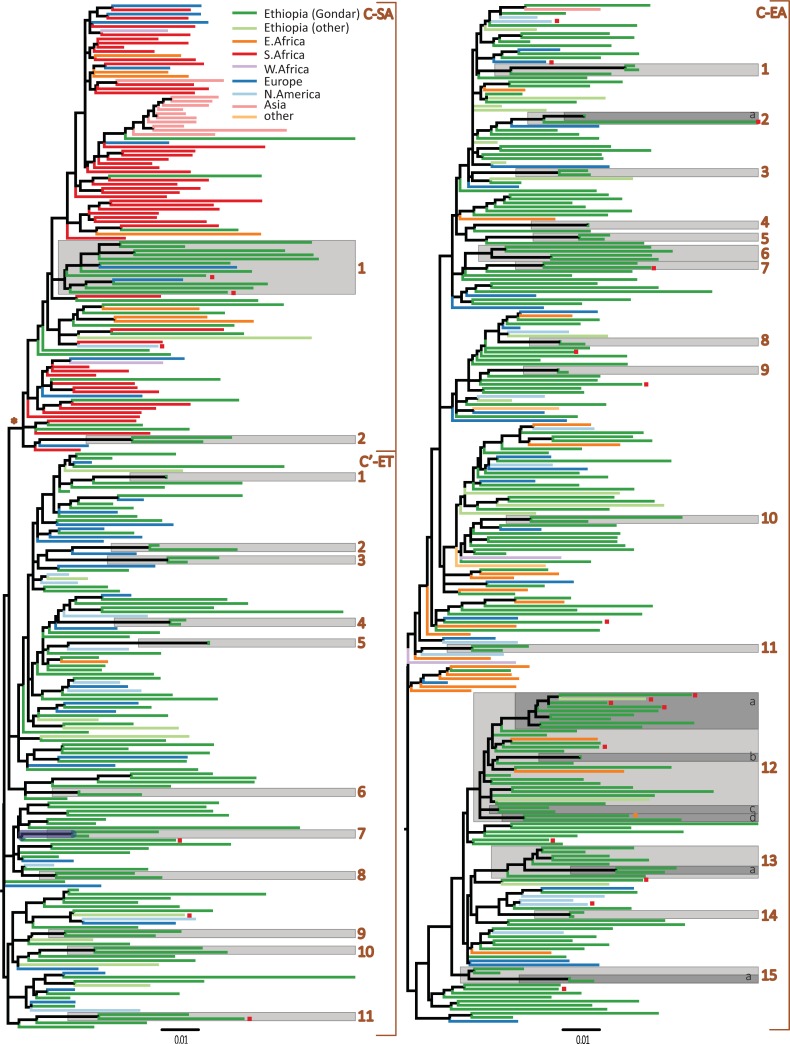
Maximum likelihood phylogenetic tree. Monophyletic clusters are shown in greyed boxes, defined as clusters with a branch support (aLRT-SH) >0.9 and with >80% sequences obtained from Gondar. Tips are coloured according to collection place. The colour code is indicated in the top of the left panel. A red square at a tip indicates a DRM sequence. (Left panel) The C-SA and C’-ET clades. The branch separating the two clades are indicated with an asterisk and is represented by a branch support of >0.9. (Right panel) The C-EA clade phylogeny.

Transmission cluster analysis was performed separately for the C-EA and C-SA/C’-ET sequences by constructing new ML trees of the two data sets (Fig 2). The C-EA phylogeny indicated that the sequences from Gondar as well as sequences from other cities in Ethiopia intermixed with the global dataset (mostly with sequences from other East African countries and Europe). The C-SA/C’-ET phylogeny showed a clear separation of the two clades similar to that seen for the subtyping phylogeny of the whole data set (S1 Fig). While the C-SA clade mostly contained sequences of southern African countries, sequences of Gondar were intermixed in the phylogeny. The C’-ET clade consisted mostly of Ethiopian, followed by sequences obtained in Europe and North America. Gondar transmission clusters were defined as statistically supported phylogenetic clusters dominated by Gondar sequences (see Methods, Table 2 and S3 Table). In total, we identified 28 clusters (which together contained 35% [105 of 301] of the sequences from Gondar) which was indicative for multiple introductions of HIV into Gondar followed by local spread (21 dyads, six medium sized clusters/networks and one large clusters; Fig 2, Table 2 and S3 Table). The Gondar C-EA sequences were found more frequently in transmission clusters compared to the C’-ET sequences (38% [68 of 177 sequences] in 15 local transmission clusters versus 23% [22 out of 97 sequences] in 11 clusters); p = 0.010, FET). Likewise, C-SA sequences were found more frequently in clusters (56% [15 of 27]) compared to C’-ET (p = 0.002, FET) while there were no difference between the number of C-EA and C-SA sequences in cluster (p = 0.098, FET). The mean cluster size for C-EA was five Gondar sequences/cluster (range: 2–28). Nine C-EA clusters had two sequences/cluster (dyads), five had ≥3–8 sequences/cluster while one cluster was large and contained 32 sequences (Fig 2). All C’-ET clusters were represented as dyads while the C-SA clusters was represented by one dyad and one cluster with 13 members. Among C-EA, six of 15 clusters contained >2 sequences which was higher than for C’-ET (none of the 11 clusters had more than two sequences; p = 0.020, FET) but this difference was not apparent between C-EA and C-SA (one cluster contained >2 sequences; p = 1.000, FET). Nine of the 15 C-EA clusters, seven of the 12 C’-ET clusters and one of the two C-SA clusters contained sequences of participants obtained in more than one of the surveys conducted in Gondar 2003–2013 indicated that clusters were populated over time in Gondar (Table 2). Moreover, eight of the 28 clusters (three C-EA clusters, three C’-ET and both of the C-SA clusters) had members from the last survey conducted 2011–2013, indicating recent transmissions within these clusters.

**Table 2:** Characteristics of Gondar transmission clusters.

	Members in Clusters						
Cluster^1^	Total	From Gondar	Outside Gondar	Collection Years	DRM^2^	tMRCA (age) ^3^	tMRCA (calender year) ^4^
C-EA-1	3	3	0	2009		30 (21–40)	1983 (1973–1992)
C-EA-2	3	3	0	2003, 2009	F53L	27 (20–37)	1986 (1976–1993)
C-EA-2a	2	2	0	2003		10 (10–12)	2003 (2001–2003)
C-EA-3	2	2	0	2009		12 (7–18)	2001 (1995–2006)
C-EA-4	2	2	0	2003		10 (10–11)	2003 (2002–2003)
C-EA-5	2	2	0	2009		10 (7–16)	2003 (1997–2006)
C-EA-6	4	4	0	2009, 2010, 2011		33 (24–44)	1980 (1969–1989)
C-EA-7	2	2	0	2003, 2009	D67E (1)	27 (19–37)	1986 (1976–1994)
C-EA-8	2	2	0	2009		8 (5–12)	2005 (2001–2008)
C-EA-9	2	2	0	2003, 2009		11 (10–12)	2002 (2001–2003)
C-EA-10	2	2	0	2003, 2009		26 (19–36)	1987 (1977–1994)
C-EA-11	2	2	0	2003, 2009		16 (12–21)	1997 (1992–2001)
C-EA-12	32	28	4	2003, 2009, 2010, 2011, 2012	G190A (4), Y181C (1)	28 (23–34)	1985 (1979–1990)
C-EA-12a	9	8	1	2003, 2009, 2010, 2011	G190A (4)	23 (19–28)	1990 (1985–1994)
C-EA-12b	2	2	0	2003, 2009		10 (10–11)	2003 (2002–2003)
C-EA-12c	2	2	0	2009, 2012	Y181C (1)	21 (16–26)	1992 (1987–1997)
C-EA-12d	2	2	0	2009		23 (17–29)	1990 (1984–1996)
C-EA-13	8	8	0	2003, 2009, 2010, 2011		26 (21–32)	1987 (1981–1992)
C-EA-13a	2	2	0	2009		13 (10–18)	2000 (1995–2003)
C-EA-14	2	2	0	2009		6 (4–9)	2007 (2004–2009)
C-EA-15	4	4	0	2003, 2009		22 (17–27)	1991 (1986–1996)
C-EA-15a	2	2	0	2009		6 (4–9)	2007 (2004–2009)
C-SA-1	13	13	0	2003, 2009, 2010, 2011, 2012,	K103N (1), G190E (1)	30 (23–39)	1983 (1974–1990)
C-SA-2	2	2	0	2011, 2012		13 (8–19)	2000 (1994–2005)
C'-ET-1	2	2	0	2009		23 (15–30)	1990 (1983–1998)
C'-ET-2	2	2	0	2010, 2012		23 (16–29)	1990 (1984–1997)
C'-ET-3	2	2	0	2009, 2010		21 (15–27)	1992 (1986–1998)
C'-ET-4	2	2	0	2009		4 (4–6)	2009 (2007–2009)
C'-ET-5	2	2	0	2009		10 (5–15)	2003 (1998–2008)
C'-ET-6	2	2	0	2003, 2009		10 (7–15)	2003 (1998–2006)
C'-ET-7	2	2	0	2003, 2010		7 (5–10)	2006 (2003–2008)
C'-ET-8	2	2	0	2010, 2012		4 (4–5)	2009 (2008–2009)
C'-ET-9	2	2	0	2010, 2011		12 (10–16)	2001 (1997–2003)
C'-ET-10	2	2	0	2009		16 (11–24)	1997 (1989–2002)
C'-ET-11	2	2	0	2003, 2009	L210W+M46I (1)	22 (17–27)	1991 (1986–1996)

^1^Cluster refers to the subtype C clades (C-EA, C-SA or C’-ET) followed by cluster number as shown in Fig 2. Clusters were defined as having an aLRT-SH support of ≥0.9 and containing at least 80% of sequences collected in Gondar.

^2^DRM: Drug resistance mutations. Mutations were defined by the Stanford Genotypic Resistance Interpretation Algorithm (http://hivdb.stanford.edu/pages/algs/HIVdb.html) using the calibrated population resistance (CPR) tool version 6.0 (http://cpr.stanford.edu/cpr/servlet/CPR), based on the WHO surveillance transmitted drug resistance mutation list of 2009.

^3^tMRCA (age): time to the most recent common ancestor. Indicated is median ages, and in parenthesis, the 95% highest posterior density credible interval. Cluster tMRCAs was determined using BEAST v1.8.4 with a logistic tree prior. Priors and other model parameters are indicated in Methods and S1 Table.

^4^tMRCA (calender year): time to the most recent common ancestor. Calendar years were obtained by subtracting the tMRCA age estimate from the most recent sampled sequence (2013) used in the analysis.

### Dating of transmission clusters of different subtype C clades in Gondar

To study the timing of introductions and differences between the different subtype C clades clusters, we performed Bayesian coalescent analysis using BEAST with sequences obtained from Gondar only (n = 301). Since the Maximum likelihood phylogeny showed that the strains of the different clades in Gondar were dispersed among sequences of other origins and most likely represented multiple introductions, the estimated root tMRCAs of each subtype C clade represent the date of the origin of the circulating subtype C clades in the region (although only Gondar sequences were used in the date estimations) while the estimated tMRCA of the transmission clusters should approximate introductions and local spread of the viral strains in Gondar, cf references [54, 62]. The age of the transmission clusters ranged from estimated dates of 1980–2009. The oldest clusters were estimated to 1980, 1983 and 1990 for C-EA, C-SA and C’-ET, respectively (Table 2 and S1 Table). Fifteen of the 28 clusters had estimated tMRCAs before year 2000. The 15 C-EA and two C-SA transmission clusters had the oldest estimated median tMRCAs (median dates: 1986 and 1991, respectively), while the 11 C’-ET clusters were younger (median date: 2001). Two C’-ET clusters were young (≤5 years) whereas the large C-EA and network C-SA clusters were estimated to be 28 and 30 years with estimated median tMRCAs of 1985 and 1983, respectively. Taken together, this indicated that the C-EA and C-SA was introduced earlier into Gondar compared to the C’-ET strain. The median population evolutionary rate of C-EA was higher than for C’-ET and C-SA (2.29 x10^-3^ and 1.85x10^-3^ versus 1.70x10^-3^ substitutions/site/year, respectively), however the 95% credible intervals overlapped (S1 Table).

### Transmission cluster associated with drug resistance mutations

Sequences with DRMs were found more frequently in clusters among the C-EA compared to the C’-ET sequences (57% versus 25%; p<0.001, FET). The C-EA Cluster 12 represented the largest cluster and contained several sub-clusters indicating a large transmission network in Gondar over an extended time period. In subcluster 12a, four of nine sequences carried the G190A mutation (Table 2 and Fig 2) which suggested cluster-associated transmission of this resistance mutation in Gondar. To verify the age estimate of cluster 12 we obtained dated phylogenies using the Bayesian MCMC method by using sequences of this cluster only in the analysis (S3 Table). The median estimated evolutionary rate of the cluster was 1.34×10^−3^ substitutions/site/year, slower than the median rate estimate of the entire C-EA cluster but with overlapping credible intervals (S1 Table). Moreover, the median tMRCA of cluster 12 was estimated to 1980 (95% HPD: 1969–1989) which was similar to the estimate (1985; 95% HPD: 1967–1985) obtained from the analysis of all Gondar sequences (Table 2 and S1 Table). Since the internode intervals in the MCMC tree represent the maximum transmission time intervals, we estimated the time between transmissions events. Analysis of the internode intervals (from the MCMC tree; S2 Fig) revealed that the maximum median transmission intervals for cluster 12 were 1.58 years (IQR: 1.24–2.31). The median parametric estimate of the population growth rate also indicated frequent transmissions associated with sequences of cluster 12 at approximately 0.5 new infections/individual/year which correspond to a median doubling time of infections every 1.34 years (16 months). The demographic plot analysis indicated that this cluster increased in size and the number of effective infections, N_e_, reached its peak in 2003, but declined thereafter (from 4990 in 2003 to 2404 in 2013; S1 Table). However, the four sequences that had the G190A mutation were collected during three of the four surveillance studies in Gondar 2003–2013 (Table 2 and Fig 2) suggesting onward transmission of this DRM over many years in Gondar.

## Discussion

This study represents the first threshold survey for DRM performed among young ART-naïve HIV-1 positive individuals in Gondar, Northern Ethiopia, using the WHO threshold methodology. We also used the sequence data obtained from drug resistance analysis together with sequence data obtained from previous studies for a detailed molecular epidemiological investigation of the HIV epidemic in Gondar. Detailed analysis stratified on the three subtype C clades identified transmission clusters in Gondar 2003–2013, which comprised 35% of all available sequences during this time. This finding indicated that HIV-1 has been introduced on multiple occasions, followed by local transmissions. Dated phylogenies revealed that about half of the local clusters originated before 2000. However, several of the clusters were long lasting and in some cases ongoing active transmission chains were detected. Importantly, we show that the G190A mutation has spread in Gondar by rapid transmission within local clusters. Even though DRM transmission within clusters has been described in other parts of the world, this is, to our knowledge, the first example of cluster-associated DRM transmission in sub Saharan Africa.

Eight to ten years after ART roll out in 2003 we found a moderate level of TDR in Gondar which is in agreement with observations of increased TDR prevalence after roll-out of ART from different world regions [5, 7, 63]. The overall prevalence of TDR (6%), found in this study is comparable to the 5.6% prevalence in sub-Saharan Africa 6–8 years after ART roll-out, and the 7.4% prevalence estimate in East Africa, eight years after ART roll-out [4, 6, 63]. Two previous studies conducted in Gondar in 2009–2010, and in the country 2009–2011, 6–8 years after ART roll out showed DRM levels of 4–6% [22, 23, 25]. Despite long term ART administration in the area there was not a significant difference in the DRM level. A direct comparison of the temporal DRM levels presented here is not possible, since the former studies targeted older age groups as opposed to our threshold study that included a younger population of HIV infected individuals. Thus, in Gondar the DRM prevalence among different age groups were not different although TDR differences between age groups and gender has been reported from other sub Saharan countries [19, 64]. However, even moderate levels of TDR among young sexually active individuals should raise concerns as it may result in less effective ART in a considerable proportion of recently HIV-1 infected individuals.

The DRMs detected in this study were all associated with the NNRTIs efavirenz (EFV) and nevirapine (NVP). This finding is not unexpected considering the low genetic barrier of these drugs to the development of resistance and their wide use as part of first line ART regimen [6]. The specific NNRTI associated mutations found in this study (K103N, G109S and Y181C) have been reported to account for the most common NNRTI-associated mutations in all world regions and HIV subtypes [65]. These mutations have also been found among patients failing treatment in Gondar, indicating a link between acquired and transmitted drug resistance [25, 66, 67]. Furthermore, strains with NNRTIs mutations may persist for a long time before being replaced by wild type making the likelihood of persistence after transmission higher [68]. In line with this, previous studies have reported significant global increases of NNRTI TDR over time since ART rollout, especially in East Africa, with an estimated 36% per year increases after ART roll out [6]. Compared to the previous studies conducted in Gondar we did not find DRMs associated with PIs and NRTIs, which could be attributed to a generally low sample size often seen in threshold studies. Thus, NNRTIs are associated with both acquired and transmitted resistance in Ethiopia, as is the case in many other LMICs, and represents an obstacle to the long-term success of ART and control of HIV transmission. As more individuals initiate ART, these observations advocate that more robust and durable first line regimens and/or improved ART monitoring are required [69].

Phylogenetic analysis revealed multiple introductions of HIV in Gondar, followed by local transmission, a phenomenon that has been described previously for other local HIV epidemics [46, 54, 70]. Our study confirms previous observations and argues against the hypothesis that a single initial lineage was introduced and became responsible for the subtype C epidemic in the country [31]. Our study also confirms pervious reports that the two subtype C clades, C-EA and C’-ET, are dominant in Ethiopia [27]. We found that the C-EA clade has been the most prevalent HIV clade in Gondar 2003–2013 since it represented the major circulating strain in Gondar at the end of the study period, as we found no evidence of significant temporal changes during this time period. However, we also show that the HIV strains of the C-SA clades, the most prevalent HIV subtype C strains prevalent in Southern Africa, have been introduced in Ethiopia, and became increasingly prevalent in Gondar 2003–2013. Sequences belonging to the C-EA clade were found to be more prevalent in clusters and were also associated with larger clusters compared to the sequences of the C’-ET clade indicating a variable intensity of the C-EA and C’-ET epidemics in Gondar. The C-EA and C’-ET strains were introduced from East Africa and Southern Africa respectively, at approximately the same time into Ethiopia (1978–1981)[30]. Our evolutionary estimates were in the range of previous estimates of subtype C and the oldest Gondar transmission clusters dated back to 1980 (C-EA), 1983 (C-SA) and 1990 (C’-ET) supporting the presence of strains of different subtype C clades at about the same time point in Gondar [30, 54]. We were able to identify old transmission clusters, since our definition of clusters followed an approach, which omitted the use of a distance cut-off, and thereby not excluding long lasting transmission chains [43, 45, 48]. Our finding showed that some clusters were long-lived and could have played an important impact in fuelling the local HIV epidemic. The larger old clusters (C-SA cluster 1 and C-EA cluster 12) had been populated until recently since the initial introduction in Gondar in the 1980s. Long-lived, large clusters have been shown to be associated with transmission of HIV in groups with high risk behaviour [43, 47]. This finding was further supported by the observation that the majority of clusters contained sequences isolated in two or more of the surveys conducted in Gondar 2003–2013. The propensity of CEA sequences to be part of clusters compared to the C’-ET could be associated with sociodemographic factors and/or underling biological differences of the CEA and C’-ET viruses. Recent infection, age, gender, drug resistance and unawareness of infection status of sexually active individuals have been shown to represent risk factors associated with transmission potential and cluster size [43, 47, 71–74]. Transmission cluster of populations with high risk of HIV acquisition are larger compared to that populations with lower risk behaviour, for example between men who have sex with men (MSM) and heterosexual networks in Europe and USA, and among recently infected individuals [43, 47, 73, 75–78]. It is also interesting to note that all transmission clusters among the C’-ET sequences represented dyads, that is, a pattern associated with less onward transmission that may represent sporadic transmission among couples and/or individuals with lower number of partners [47]. Since we had no data regarding risk behaviours of the study participants, further studies are needed to establish any association between risk behaviours and gender as well as other sociodemographic factors with clustering of the different subtype C strains in Ethiopia. Moreover, it has previously been suggested that biological differences between the two Ethiopian strains may be coupled to transmissibility due to a generally higher viral load among patients infected with C’-ET which has been suggested as an explanation for an observed abundance of the C’-ET strain in Ethiopia in previous studies [61, 79]. However, our results indicated a higher prevalence of C-EA in Gondar 2003–2013 and further studies are needed to establish any association between biological properties, replicative capacity, disease progression, risk behaviours and transmission rates among the strains of the Ethiopian subtype C clades [79, 80]

Besides being associated with more sequences and larger clusters, the CEA clade was also associated with clustering of DRM sequences. The role of DRMs in clustered transmission has been addressed in a number of studies. Previous studies of the Swiss HIV cohort, indicated that TDR viruses were more associated with clustering compared to non-TDR viruses [73]. An association between clustering and increased transmission of viruses harbouring NNRTI DRMs, including the G190A DRM found here, has been described in Quebec, Canada. Transmission clustering were also associated with sexual behaviour, mainly that of MSM [19]. In the present study, we identified one cluster with the G190A mutation with onward transmissions of this DRM for at least 8 years (2003–2010). The G190A mutation represents a slowly reverting HIVDR mutation and when such mutations are present in a population with frequent transmission it might persist and expand [67, 81]. We estimated that the entire large cluster and the G190A cluster had transmission intervals at least every year. Since not all members of a given transmission cluster have been sampled, the average time between transmissions is overestimated since the transmission chain is incomplete, suggesting that transmissions have taken place at a much higher rate [82]. In fact, many molecular epidemiology studies suffer from low sampling, representing only a fraction of all infected individuals at the time of the study. The 301 sequences from Gondar (population: ~207,000) 2003–2013 were estimated to roughly represent 1.3% of the infected population during the study period (considering a mean prevalence of 10.6% 2003–2014; [32] and Methods). Using a linear projection the cluster could be up to 75 times larger, i.e cluster 12 with 32 individual specimens may represent 2400 individual or more in the cluster (which could be compared with the estimated number of effective infections in 2013 of 2400 of the demographic plot analysis; S1 Table) and the G190A associated subcluster 12a of about 450 individuals. Although these numbers represent a rough estimate it illustrates the impact of fast onward transmission that is commonly seen among groups with recent infections and/or with high risk of HIV acquisitions. Fast transmission times may favour the accumulation of TDR mutations especially when transmission times are faster than the average time of reversions of the mutations.

In conclusion, our study showed a moderate prevalence of TDR in Gondar. We could also demonstrate a hitherto unrecognized diversity of the HIV-1 epidemic in the area, and confirm pervious reports that the two subtype C clades, C-EA and C’-ET, are dominant in Ethiopia but our study showed an increased diversity in the epidemic since we showed multiple introductions and that viral strains of the C-SA clade, prevalent in southern Africa, has been introduced and increased in prevalence in Gondar 2003–2013. We also show differences in transmission clusters sizes of the different subtype C clades and association with DRMs. These findings provide epidemiological insights not available using standard surveillance approaches and may inform the improvement of public health strategies in HIV prevention in Ethiopia and similar LMIC settings.

## Supporting information

S1 Text HIV-1 subtyping, identification of intra-subtype recombinants and construction of a reference data set. (DOCX)

S1 Table Population dynamics and evolutionary estimates of subtype C clades in Gondar. (DOCX)

S2 Table Taxa included in the phylogenetic analysis. (DOCX)

S3 Table Taxa associated with clusters. (DOCX)

S1 Fig Phylogenetic subtyping of sequences obtained from Gondar 2003–2013. (DOCX)

S2 Fig Maximum clade credibility tree of C-EA Cluster 12. (DOCX)
