# Supplementary material for: HIV-genetic diversity and drug resistance transmission clusters in Gondar, Northern Ethiopia, 2003-2013
Source: PLoS One. 2018 Oct 10;13(10):e0205446. doi: 10.1371/journal.pone.0205446 (PMC6179264; doi:10.1371/journal.pone.0205446)
Supplement: S1 Text — (DOCX) [file pone.0205446.s001.docx]

**S1 Text**

**HIV-1 subtyping**

A 1084-bp fragment of HIV-1 pol comprising amino acids 6-99 of the protease (PR) and 1-251 of the reverse transcriptase (RT) was amplified using an in-house genotyping assay as described [[1](#_ENREF_1)]. Total HIV-1 RNA was extracted from 140µl patient plasma using QIAamp Viral RNA Mini kit (Qiagen, Hilden, Germany) according to the manufacturers instructions and 10μl of extracted nucleic acids was added to a 40 μl RT-PCR master mix containing SuperScriptTM III one step RT-PCR enzyme with Platinum TaqDNA polymerase high fidelity. Then 2μl of the first round reverse transcription polymerase chain reaction product was added to 48μl PCR mix containing primers and AmpliTaq gold LD DNA polymerase (Applied Biosystems, Foster City, CA). The primers used for the one step RT-PCR were PRTM-F1 and RT-R1 and RT-R2 and PRt-F2 were used for the nested PCR [[1](#_ENREF_1)]. Cycling conditions for the reverse transcription polymerase chain reaction was 45 minute at 50°C and 2 minute at 94°C followed by 40 cycles of 15 sec at 94°C, 20 sec at 50°C, and 2 min at 72°C followed by extension at 72°C for 10 minutes. Cycling conditions for the nested polymerase chain reaction was 4 minute at 94ºC followed by 40 cycles of 15 sec at 94°C, 20 sec at 55°C, and 2 min at 72°C followed by extension at 72^o^c for 10 minute.

The PCR products were electrophoresed on 1% agarose gel electrophoresis to check the yield, and were then purified with QIAquick PCR Purification Kit (Qiagen, Hilden, Germany) according to the manufacturers recommendations.

A premixed Big Dye terminator sequencing reagents (Applied Biosystems, Foster City, CA), were used to sequence the PCR products with 6 in house primers on both strands [[1](#_ENREF_1)]. ABI 3100 and ABI 3500xl Genetic Analyzer were used for sequencing. Sequence assembly and editing were performed using Standalone RECall V 2.0 HIV-1 sequencing analysis tool (REF).

**Identification of intra-subtype recombinants**

Previous studies have shown that two distinct subtype C strains are co-circulating in Ethiopia, designated C and C’ [[2](#_ENREF_2),[3](#_ENREF_3)], and recombinant forms between the C clades [[4](#_ENREF_4)]. Further molecular characterization revealed that the Ethiopian C strain were similar to strains circulating in other East African countries while the C’ strain were more closely related to the strains found in countries in Southern Africa. Ten distinct subype C clades (termed C1-C10) have been defined based on phylogenetic relationship by Delatorre and Bello [[5](#_ENREF_5)]. Clades C1-C9 were mainly represented by sequences obtained from countries in southern Africa while the C10 clade represented sequences from East and Central Africa, cf Table 1 in [[6](#_ENREF_6)]. The C10 clade corresponded to the East African (C-EA) clade of Delatorre and Bello, which includes the Ethiopian C strain. The Ethiopian C’ clade (C’-ET) represented a distinct subclade of the major Southern African clades. Thomson and Fernández-García classified the C’ as belonging to the Southern African C9 clade but it forms a unique phylogenetic distinct subcluster (see supplementary figure 5 in [[6](#_ENREF_6)], and Fig. S1_1). Thus, based on previous studies, the major African subtype C stains can be divided into three distinct phylogenetic groups: the southern African subtype C clades (C-SA), the Ethiopian C’ clade (C’-ET), and the central and east African subtype C clade (C-EA).

**Construction of a reference data set**

To identify putative intra-subtype C recombinants we first constructed an non-recombinant reference data set of the different subtype C clades which were obtained from Thomson and Fernández-García (Fig.3 and Supplementary Fig. 5 in [[6](#_ENREF_6)]) and Delatorre and Bello (supplementary figure 3 in [[5](#_ENREF_5)]), shown in Table S1_1. The polymerase region (corresponding to pos 2248-3309 of HXB2, Genbank Accession Number K03455) was retrieved. Before conducting the phylogenetic analysis, the data set was screened for putative recombinant sequences using an iterative version of the phi-test and RDP v.3.44 [[7](#_ENREF_7),[8](#_ENREF_8)]. A maximum likelihood phylogenetic trees was constructed using Garli v2.0 with the GTR+I+Γ substitution model [[9](#_ENREF_9)]. Branch support was obtained using the aLRT-SH test implemented in PhyML v3.1 [[10](#_ENREF_10)]. An aLRT-SH value of ≥0.9 was considered significant [[11](#_ENREF_11),[12](#_ENREF_12)]. The phylogenetic tree clearly indicated that the sequences were divided into distinct clades (Fig.S1_1).

The clade sequences indicated in Table S1_1 and Fig. S1_1 were used to construct a scoring matrix as described in the jpHMM documentation (http://jphmm.gobics.de/; [[13](#_ENREF_13)]). For the other HIV-1 subtypes we used polymerase sequences derived from the HIV reference data set from Los Alamos HIV sequence database (http://www.hiv.lanl.gov). Three training sets were used to optimize and validate the jpHMM parameter settings: 1) an artificial data set containing chimeras of polymerase sequences derived from a combination of clade specific sequences were assembled, such that the first 340-bp of the sequence contained a C-EA sequences, the middle 340-bp part were made up of a C’-ET sequence while the last 340-bp were derived from a C-EA sequence. Twenty different chimeric sequences with different compositions and combinations of clade fragments were created and screened using jpHMM with different jump (j) and beam-width (bw) parameters. A bw =1e-10 and a j=9.5 e-03 correctly identified these artificial chimeras as recombinant sequences; 2) Using the same setting we also screened a number of previously identified intra-subtype C recombinants (C-EA/C’-ET recombinants) [[4](#_ENREF_4),[5](#_ENREF_5)]. In all cases, these sequences were identified as recombinant sequences; 3) We screened the reference sequences to its own matrix with addition of reference sequences using the above parameters and in all cases, the correct subtype and clade identity was found (command line: jpHMM -s FileWithSequencesToScreen.fas -v HIV -Q blat -B 1e-10 -j 9.5e-03, where –v HIV represents the custom made scoring matrix of subtype C clades and HIV-1 subtype reference sequences). Finally, we screened the entire data set for the analysis, shown in main manuscript Fig 2 and S2 Fig. All sequences that were identified as putative recombinant sequences were further analysed using Simplot v3.5.1 and phylogenetic analysis. If the Simplot analysis indicated that a sequence represented a putative recombinant, phylogenetic analysis were performed using the fragments indicated as belonging to different subtype C clades of the putative recombinant sequence. Both the Simplot and phylogenetic analyses used a background of reference sequences.

**Figures**


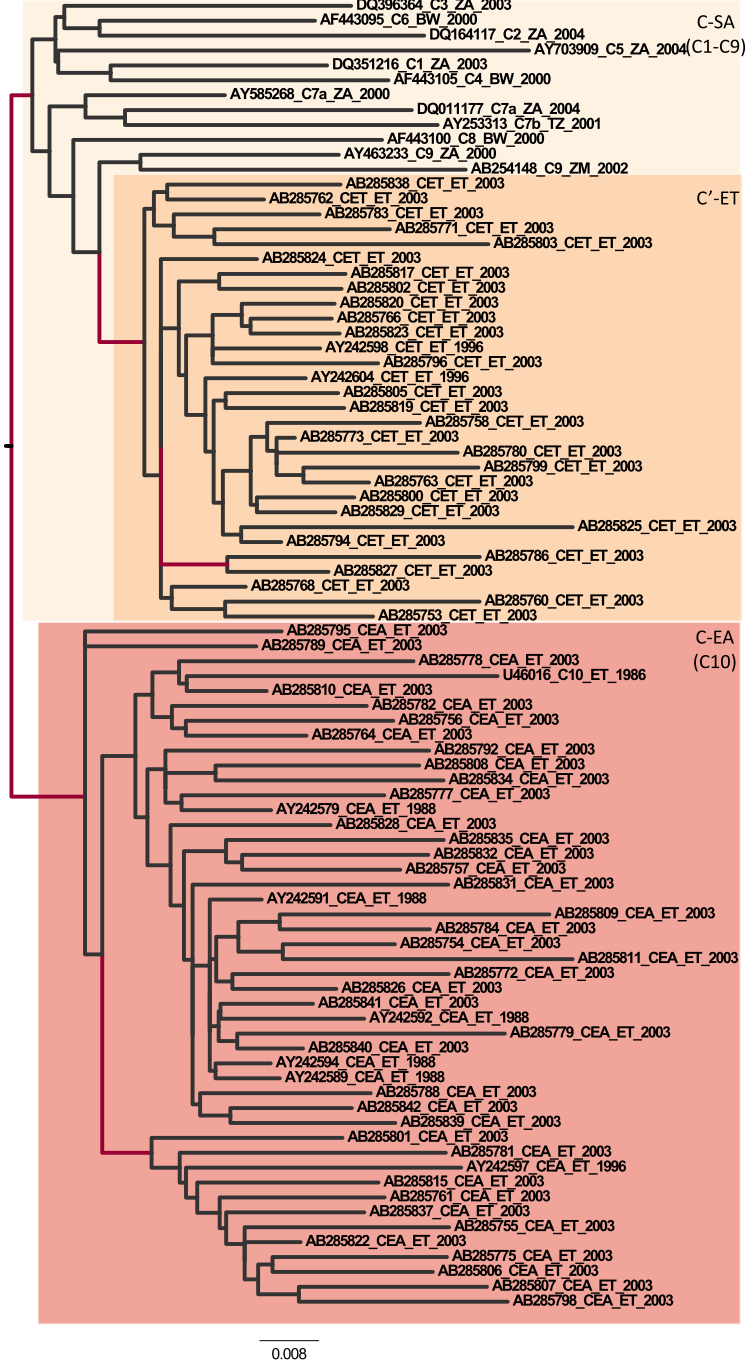


Figure S1_1. Maximum likelihood phylogenetic tree of the reference data set, derived from Garli v2.0. Significantly supported branches are highlighted in red (aLRT-SH≥0.9). Each supported group of sequences (C-SA, C’-ET and C-EA clades) is highlighted in a coloured box.

**Table SI_1.** Nucleotide sequences used in the assignment to different subtype C clades

| **Genbank Accession Number** | **Clade^1^** | **Country^2^** | **Year^3^** |  | **Genbank Accession Number** | **Clade^1^** | **Country^2^** | **Year^3^** |
| --- | --- | --- | --- | --- | --- | --- | --- | --- |
| AB285754 | C-EA | ET | 2003 |  | AB285753 | C'-ET | ET | 2003 |
| AB285755 | C-EA | ET | 2003 |  | AB285758 | C'-ET | ET | 2003 |
| AB285756 | C-EA | ET | 2003 |  | AB285760 | C'-ET | ET | 2003 |
| AB285757 | C-EA | ET | 2003 |  | AB285762 | C'-ET | ET | 2003 |
| AB285761 | C-EA | ET | 2003 |  | AB285763 | C'-ET | ET | 2003 |
| AB285764 | C-EA | ET | 2003 |  | AB285766 | C'-ET | ET | 2003 |
| AB285772 | C-EA | ET | 2003 |  | AB285768 | C'-ET | ET | 2003 |
| AB285775 | C-EA | ET | 2003 |  | AB285771 | C'-ET | ET | 2003 |
| AB285777 | C-EA | ET | 2003 |  | AB285773 | C'-ET | ET | 2003 |
| AB285778 | C-EA | ET | 2003 |  | AB285780 | C'-ET | ET | 2003 |
| AB285779 | C-EA | ET | 2003 |  | AB285783 | C'-ET | ET | 2003 |
| AB285782 | C-EA | ET | 2003 |  | AB285786 | C'-ET | ET | 2003 |
| AB285784 | C-EA | ET | 2003 |  | AB285794 | C'-ET | ET | 2003 |
| AB285788 | C-EA | ET | 2003 |  | AB285796 | C'-ET | ET | 2003 |
| AB285789 | C-EA | ET | 2003 |  | AB285799 | C'-ET | ET | 2003 |
| AB285792 | C-EA | ET | 2003 |  | AB285800 | C'-ET | ET | 2003 |
| AB285798 | C-EA | ET | 2003 |  | AB285802 | C'-ET | ET | 2003 |
| AB285806 | C-EA | ET | 2003 |  | AB285803 | C'-ET | ET | 2003 |
| AB285807 | C-EA | ET | 2003 |  | AB285805 | C'-ET | ET | 2003 |
| AB285808 | C-EA | ET | 2003 |  | AB285817 | C'-ET | ET | 2003 |
| AB285809 | C-EA | ET | 2003 |  | AB285819 | C'-ET | ET | 2003 |
| AB285810 | C-EA | ET | 2003 |  | AB285820 | C'-ET | ET | 2003 |
| AB285811 | C-EA | ET | 2003 |  | AB285823 | C'-ET | ET | 2003 |
| AB285815 | C-EA | ET | 2003 |  | AB285824 | C'-ET | ET | 2003 |
| AB285822 | C-EA | ET | 2003 |  | AB285827 | C'-ET | ET | 2003 |
| AB285826 | C-EA | ET | 2003 |  | AB285829 | C'-ET | ET | 2003 |
| AB285828 | C-EA | ET | 2003 |  | AB285838 | C'-ET | ET | 2003 |
| AB285831 | C-EA | ET | 2003 |  | AY242598 | C'-ET | ET | 1996 |
| AB285832 | C-EA | ET | 2003 |  | AY242604 | C'-ET | ET | 1996 |
| AB285834 | C-EA | ET | 2003 |  | AB254148 | C-SA (C9) | ZM | 2002 |
| AB285835 | C-EA | ET | 2003 |  | AF067155 | C-SA | IN | 1995 |
| AB285837 | C-EA | ET | 2003 |  | AF443095 | C-SA (C6) | BW | 2000 |
| AB285839 | C-EA | ET | 2003 |  | AF443100 | C-SA (C8) | BW | 2000 |
| AB285840 | C-EA | ET | 2003 |  | AF443105 | C-SA (C4) | BW | 2000 |
| AB285841 | C-EA | ET | 2003 |  | AY253313 | C-SA (C7b) | TZ | 2001 |
| AB285842 | C-EA | ET | 2003 |  | AY463233 | C-SA (C9) | ZA | 2000 |
| AY242579 | C-EA | ET | 1988 |  | AY585268 | C-SA (C7a) | ZA | 2000 |
| AY242589 | C-EA | ET | 1988 |  | AY703909 | C-SA (C5) | ZA | 2004 |
| AY242591 | C-EA | ET | 1988 |  | DQ011177 | C-SA (C7a) | ZA | 2004 |
| AY242592 | C-EA | ET | 1988 |  | DQ164117 | C-SA (C2) | ZA | 2004 |
| AY242594 | C-EA | ET | 1988 |  | DQ351216 | C-SA (C1) | ZA | 2003 |
| AY242597 | C-EA | ET | 1996 |  | DQ396364 | C-SA (C3) | ZA | 2003 |
| U46016 | C-EA (C10) | ET | 1986 |  | U52953 | C-SA | BR | 1992 |

Footnote to Table SI1.

**^2^**Reference sequences defined in [[5](#_ENREF_5),[6](#_ENREF_6)]; C-EA: East African subtype C clade; C'-ET: Ethiopian subtype C clade; C-SA: Southern African subtype C clades,

**^2^**Two-letter country code (iso 3166-2); https://datahub.io/core/country-list

**^2^**Year of sample collection

**References**

1. Zhou Z, Wagar N, DeVos JR, Rottinghaus E, Diallo K, et al. (2011) Optimization of a low cost and broadly sensitive genotyping assay for HIV-1 drug resistance surveillance and monitoring in resource-limited settings. PLoS One 6: e28184.

2. Abebe A, Kuiken CL, Goudsmit J, Valk M, Messele T, et al. (1997) HIV type 1 subtype C in Addis Ababa, Ethiopia. AIDS Res Hum Retroviruses 13: 1071-1075.

3. Abebe A, Pollakis G, Fontanet AL, Fisseha B, Tegbaru B, et al. (2000) Identification of a genetic subcluster of HIV type 1 subtype C (C') widespread in Ethiopia. AIDS Res Hum Retroviruses 16: 1909-1914.

4. Pollakis G, Abebe A, Kliphuis A, De Wit TF, Fisseha B, et al. (2003) Recombination of HIV type 1C (C'/C") in Ethiopia: possible link of EthHIV-1C' to subtype C sequences from the high-prevalence epidemics in India and Southern Africa. AIDS Res Hum Retroviruses 19: 999-1008.

5. Delatorre EO, Bello G (2012) Phylodynamics of HIV-1 subtype C epidemic in east Africa. PLoS One 7: e41904.

6. Thomson MM, Fernandez-Garcia A (2011) Phylogenetic structure in African HIV-1 subtype C revealed by selective sequential pruning. Virology 415: 30-38.

7. Bruen TC, Philippe H, Bryant D (2006) A simple and robust statistical test for detecting the presence of recombination. Genetics 172: 2665-2681.

8. Martin DP, Lemey P, Lott M, Moulton V, Posada D, et al. (2010) RDP3: a flexible and fast computer program for analyzing recombination. Bioinformatics 26: 2462-2463.

9. Zwickl DJ (2006) Genetic algorithm approaches for the phylogenetic analysis of large biological sequence datasets under the maximum likelihood criterion: The University of Texas at Austin.

10. Guindon S, Dufayard JF, Lefort V, Anisimova M, Hordijk W, et al. (2010) New algorithms and methods to estimate maximum-likelihood phylogenies: assessing the performance of PhyML 3.0. Syst Biol 59: 307-321.

11. Anisimova M, Gil M, Dufayard JF, Dessimoz C, Gascuel O (2011) Survey of branch support methods demonstrates accuracy, power, and robustness of fast likelihood-based approximation schemes. Syst Biol 60: 685-699.

12. Guindon S, Gascuel O (2003) A simple, fast, and accurate algorithm to estimate large phylogenies by maximum likelihood. Syst Biol 52: 696-704.

13. Schultz AK, Zhang M, Leitner T, Kuiken C, Korber B, et al. (2006) A jumping profile Hidden Markov Model and applications to recombination sites in HIV and HCV genomes. BMC Bioinformatics 7: 265.
