# Supplementary material for: HIV-genetic diversity and drug resistance transmission clusters in Gondar, Northern Ethiopia, 2003-2013
Source: PLoS One. 2018 Oct 10;13(10):e0205446. doi: 10.1371/journal.pone.0205446 (PMC6179264; doi:10.1371/journal.pone.0205446)
Supplement: S1 Table — (DOCX) [file pone.0205446.s002.docx]

| **S1 Table. Population dynamics and evolutionary estimates for subtype C clades in Gondar** | | | |  |
| --- | --- | --- | --- | --- |
|  | Subtype C clade/cluster | | | |
|  | **C-SA** | **C'-ET** | **C-EA** | **C-EA (Cluster 12)** |
| No. of samples from Gondar | 27 | 97 | 177 | 28 |
| Range of collection (years) | 2003-2013 | 1988-2013 | 2003-2013 | 2000-2013 |
| Rate prior ^1^ | 2.15 (1.79-2.60) | 1.44 (0.97-1.91) | 1.26 (0.88-1.64) | 1.00 (0.59-3.00) |
| Best clock model | strict | relaxed | relaxed | relaxed |
| Best demographic model | logistic/skyride | logistic | logistic | logistic/skyride |
| Median evolutionary substitution rate (95% HPD)^2^ | 1.70 (1.22-2.22) | 1.85 (1.52-2.19) | 2.29 (1.57-2.99) | 1.34 (0.79-1.91) |
| year when N_e_ reached maximum; effective no. of infections, N_e_ (95% HPD)^3^ | 2013; 4181 (769-58334) | 2001; 9923 (3067-42778) | 1997; 26571 (11776-132047) | 2003; 4990 (1268-32753) |
| Effective no. of Infections, N_e_, at last sampling year, 2013 (95% HPD) | 4181 (769-58334) | 1835 (358-13680) | 16501 (4715-84857) | 2404 (467-28385) |
| Median age of clusters (range or IQR) | 22 (range: 13-30) | 12 (IQR: 7-22) | 27 (IQR: 10-31) | 33 (IQR: 24-42) |
| Median age (calendar years) of clusters (range or IQR) | 1991 (range: 1983-2000) | 2001 (IQR: 1991-2006) | 1986 (IQR: 1982-2003) | 1980 (IQR: 1971-1989) |
| Median year of first Gondar cluster (95% HPD) | 1983 (1974-1990) | 1990 (1983-1998) | 1980 (1969-1989) | n.a |
| Median rate of population growth (95% HPD)^4^ | 0.68 (0.37-1.09) | 0.58 (0.41-0.79) | 0.43 (0.28-0.60) | 0.52 (0.25-0.84) |
| Median epidemic doubling time (years) (95% HPD)^5^ | 1.02 (0.64-1.88) | 1.19 (0.88-1.69) | 1.60 (1.16-2.46) | 1.34 (0.83-2.76) |

^1^ Median number of substitutions/site/year x 10^-3^; The rate prior was determined from a global data set, as described in Methods, or obtained from the literature (for C-SA, ref [54])
^2^ Median number of substitutions/site/year x 10^-3^. The rates were obtained using sequences from Gondar only with the rate priors indicated and a logistic tree prior, as described in Methods.
^3^ N_e_ (number of effective infections) which reflects the number of infections contribution to new infections
^4^ Population growth rate (r), as median number of new infections/individual/year, determined in BEAST v1.8.4 using a logistic tree prior.
^5^ The time (years) required to double the effective number of infections (λ), calculated as λ = ln(2)/r, where r is the population growth rate.
