# Supplementary material for: HIV-genetic diversity and drug resistance transmission clusters in Gondar, Northern Ethiopia, 2003-2013
Source: PLoS One. 2018 Oct 10;13(10):e0205446. doi: 10.1371/journal.pone.0205446 (PMC6179264; doi:10.1371/journal.pone.0205446)
Supplement: S2 Table — (DOCX) [file pone.0205446.s003.docx]

| **S2 Table**. Taxa included in the phylogenetic analysis | | | | |  |  |
| --- | --- | --- | --- | --- | --- | --- |
| **Taxa** | **DR** | **Country** | **City ET** | **Collection Year** | **Subtype/Clade** |  |
| AB285754 | N | ET | Gondar | 2003 | C-EA |  |
| AB285755 | N | ET | Gondar | 2003 | C-EA |  |
| AB285756 | N | ET | Gondar | 2003 | C-EA |  |
| AB285757 | N | ET | Gondar | 2003 | C-EA |  |
| AB285761 | N | ET | Gondar | 2003 | C-EA |  |
| AB285764 | N | ET | Gondar | 2003 | C-EA |  |
| AB285769 | N | ET | Gondar | 2003 | C-EA |  |
| AB285770 | N | ET | Gondar | 2003 | C-EA |  |
| AB285772 | N | ET | Gondar | 2003 | C-EA |  |
| AB285774 | N | ET | Gondar | 2003 | C-EA |  |
| AB285775 | N | ET | Gondar | 2003 | C-EA |  |
| AB285776 | N | ET | Gondar | 2003 | C-EA |  |
| AB285777 | N | ET | Gondar | 2003 | C-EA |  |
| AB285778 | N | ET | Gondar | 2003 | C-EA |  |
| AB285779 | N | ET | Gondar | 2003 | C-EA |  |
| AB285781 | N | ET | Gondar | 2003 | C-EA |  |
| AB285782 | N | ET | Gondar | 2003 | C-EA |  |
| AB285784 | N | ET | Gondar | 2003 | C-EA |  |
| AB285787 | N | ET | Gondar | 2003 | C-EA |  |
| AB285788 | N | ET | Gondar | 2003 | C-EA |  |
| AB285789 | N | ET | Gondar | 2003 | C-EA |  |
| AB285791 | N | ET | Gondar | 2003 | C-EA |  |
| AB285792 | N | ET | Gondar | 2003 | C-EA |  |
| AB285795 | N | ET | Gondar | 2003 | C-EA |  |
| AB285798 | D | ET | Gondar | 2003 | C-EA |  |
| AB285801 | N | ET | Gondar | 2003 | C-EA |  |
| AB285804 | N | ET | Gondar | 2003 | C-EA |  |
| AB285806 | N | ET | Gondar | 2003 | C-EA |  |
| AB285807 | D | ET | Gondar | 2003 | C-EA |  |
| AB285808 | N | ET | Gondar | 2003 | C-EA |  |
| AB285809 | N | ET | Gondar | 2003 | C-EA |  |
| AB285810 | N | ET | Gondar | 2003 | C-EA |  |
| AB285811 | N | ET | Gondar | 2003 | C-EA |  |
| AB285814 | N | ET | Gondar | 2003 | C-EA |  |
| AB285815 | N | ET | Gondar | 2003 | C-EA |  |
| AB285816 | N | ET | Gondar | 2003 | C-EA |  |
| AB285821 | N | ET | Gondar | 2003 | C-EA |  |
| AB285822 | N | ET | Gondar | 2003 | C-EA |  |
| AB285826 | N | ET | Gondar | 2003 | C-EA |  |
| AB285828 | N | ET | Gondar | 2003 | C-EA |  |
| AB285831 | N | ET | Gondar | 2003 | C-EA |  |
| AB285832 | N | ET | Gondar | 2003 | C-EA |  |
| AB285833 | N | ET | Gondar | 2003 | C-EA |  |
| AB285834 | N | ET | Gondar | 2003 | C-EA |  |
| AB285835 | N | ET | Gondar | 2003 | C-EA |  |
| AB285837 | N | ET | Gondar | 2003 | C-EA |  |
| AB285839 | N | ET | Gondar | 2003 | C-EA |  |
| AB285840 | N | ET | Gondar | 2003 | C-EA |  |
| AB285841 | N | ET | Gondar | 2003 | C-EA |  |
| AB285842 | N | ET | Gondar | 2003 | C-EA |  |
| AB285843 | N | ET | Gondar | 2003 | C-EA |  |
| AF447844 | N | UG |  | 1990 | C-EA |  |
| AJ419466 | N | DK |  | 2000 | C-EA |  |
| AJ583722 | N | SN |  | 1998 | C-EA |  |
| AM260222 | N | BI |  | 2002 | C-EA |  |
| AM260241 | N | BI |  | 2002 | C-EA |  |
| AM260257 | N | BI |  | 2002 | C-EA |  |
| AM260267 | N | BI |  | 2002 | C-EA |  |
| AM260320 | N | BI |  | 2002 | C-EA |  |
| AY102486 | N | SD |  | 1998 | C-EA |  |
| AY102489 | N | SD |  | 1999 | C-EA |  |
| FDR056 | N | SD |  | 1999 | C-EA |  |
| AY102497 | N | SD |  | 1999 | C-EA |  |
| AY102498 | N | SD |  | 1999 | C-EA |  |
| AY102502 | N | SD |  | 1999 | C-EA |  |
| AY165219 | N | SE |  | 1999 | C-EA |  |
| AY165225 | N | SE |  | 2000 | C-EA |  |
| AY242579* | N | ET | Addis Ababa | 1988 | C-EA |  |
| AY242588 | N | ET | Jimma | 1996 | C-EA |  |
| AY242589* | N | ET | Dire Dawa | 1988 | C-EA |  |
| AY242591* | N | ET | Dire Dawa | 1988 | C-EA |  |
| AY242592* | N | ET | Assab | 1988 | C-EA |  |
| AY242594* | N | ET | Dessie | 1988 | C-EA |  |
| AY242597 | N | ET | Dessie | 1996 | C-EA |  |
| AY255823 | N | IL |  | 1999 | C-EA |  |
| AY371691 | N | ER |  | 2000 | C-EA |  |
| AY372165 | N | IT |  | 2000 | C-EA |  |
| DQ113165 | N | CU |  | 2003 | C-EA |  |
| DR011 | N | ET | Gondar | 2011 | C-EA |  |
| DR015 | N | ET | Gondar | 2011 | C-EA |  |
| DR029 | N | ET | Gondar | 2011 | C-EA |  |
| DR038 | N | ET | Gondar | 2011 | C-EA |  |
| DR045 | N | ET | Gondar | 2011 | C-EA |  |
| DR047 | N | ET | Gondar | 2011 | C-EA |  |
| DR052 | N | ET | Gondar | 2011 | C-EA |  |
| DR056 | D | ET | Gondar | 2012 | C-EA |  |
| DR063 | N | ET | Gondar | 2012 | C-EA |  |
| DR066 | N | ET | Gondar | 2012 | C-EA |  |
| DR068 | N | ET | Gondar | 2012 | C-EA |  |
| DR072 | N | ET | Gondar | 2012 | C-EA |  |
| DR073 | N | ET | Gondar | 2012 | C-EA |  |
| DR078 | N | ET | Gondar | 2012 | C-EA |  |
| DR079 | N | ET | Gondar | 2012 | C-EA |  |
| DR080 | N | ET | Gondar | 2012 | C-EA |  |
| DR082 | N | ET | Gondar | 2012 | C-EA |  |
| DR084 | N | ET | Gondar | 2012 | C-EA |  |
| DR086 | N | ET | Gondar | 2012 | C-EA |  |
| DR088 | D | ET | Gondar | 2012 | C-EA |  |
| DR089 | N | ET | Gondar | 2012 | C-EA |  |
| DR094 | N | ET | Gondar | 2013 | C-EA |  |
| DR507 | N | ET | Gondar | 2011 | C-EA |  |
| DR508 | N | ET | Gondar | 2011 | C-EA |  |
| DR510 | N | ET | Gondar | 2011 | C-EA |  |
| DR511 | N | ET | Gondar | 2011 | C-EA |  |
| DR513 | N | ET | Gondar | 2011 | C-EA |  |
| DR514 | N | ET | Gondar | 2011 | C-EA |  |
| DR519 | N | ET | Gondar | 2012 | C-EA |  |
| DR527 | N | ET | Gondar | 2012 | C-EA |  |
| EU611405 | N | ?? |  | 2003 | C-EA |  |
| EU693681 | N | US |  | 1998 | C-EA |  |
| EU693892 | N | US |  | 1997 | C-EA |  |
| GQ398881 | N | NO |  | 2003 | C-EA |  |
| GQ399394 | N | NO |  | 2005 | C-EA |  |
| GQ400400 | N | NO |  | 2003 | C-EA |  |
| GQ400501 | N | DK |  | 2003 | C-EA |  |
| GQ400508 | N | NL |  | 2003 | C-EA |  |
| GQ400950 | N | SE |  | 2003 | C-EA |  |
| HM191524 | N | RO |  | 2005 | C-EA |  |
| JF769782 | N | CH |  | 2003 | C-EA |  |
| JQ071491 | N | TZ |  | 2004 | C-EA |  |
| JQ698845 | N | SE |  | 2005 | C-EA |  |
| JX299585 | N | SE |  | 2007 | C-EA |  |
| JX459980 | N | US |  | 2006 | C-EA |  |
| JX460007 | N | US |  | 2007 | C-EA |  |
| JX460026 | N | US |  | 2007 | C-EA |  |
| JX460186 | N | US |  | 2008 | C-EA |  |
| JX460226 | N | US |  | 2009 | C-EA |  |
| JX460408 | D | US |  | 2010 | C-EA |  |
| JX460736 | D | US |  | 2011 | C-EA |  |
| KF026059 | N | ET | Gondar | 2009 | C-EA |  |
| KF026060 | N | ET | Gondar | 2009 | C-EA |  |
| KF026061 | N | ET | Gondar | 2009 | C-EA |  |
| KF026064 | N | ET | Gondar | 2009 | C-EA |  |
| KF026065 | N | ET | Gondar | 2009 | C-EA |  |
| KF026068 | D | ET | Gondar | 2009 | C-EA |  |
| KF026069 | N | ET | Gondar | 2009 | C-EA |  |
| KF026072 | N | ET | Gondar | 2009 | C-EA |  |
| KF026073 | D | ET | Gondar | 2009 | C-EA |  |
| KF026074 | N | ET | Gondar | 2009 | C-EA |  |
| KF026075 | N | ET | Gondar | 2009 | C-EA |  |
| KF026076 | N | ET | Gondar | 2009 | C-EA |  |
| KF026077 | N | ET | Gondar | 2009 | C-EA |  |
| KF026079 | N | ET | Gondar | 2009 | C-EA |  |
| KF026080 | N | ET | Gondar | 2009 | C-EA |  |
| KF026082 | N | ET | Gondar | 2009 | C-EA |  |
| KF026083 | N | ET | Gondar | 2009 | C-EA |  |
| KF026092 | N | ET | Gondar | 2009 | C-EA |  |
| KF026093 | N | ET | Gondar | 2009 | C-EA |  |
| KF026097 | N | ET | Gondar | 2009 | C-EA |  |
| KF026098 | D | ET | Gondar | 2009 | C-EA |  |
| KF026102 | N | ET | Gondar | 2009 | C-EA |  |
| KF026103 | N | ET | Gondar | 2009 | C-EA |  |
| KF026104 | N | ET | Gondar | 2009 | C-EA |  |
| KF026106 | N | ET | Gondar | 2009 | C-EA |  |
| KF026107 | N | ET | Gondar | 2009 | C-EA |  |
| KF026109 | N | ET | Gondar | 2009 | C-EA |  |
| KF026110 | N | ET | Gondar | 2009 | C-EA |  |
| KF026111 | N | ET | Gondar | 2009 | C-EA |  |
| KF026113 | N | ET | Gondar | 2009 | C-EA |  |
| KF026114 | N | ET | Gondar | 2009 | C-EA |  |
| KF026115 | N | ET | Gondar | 2009 | C-EA |  |
| KF026116 | N | ET | Gondar | 2009 | C-EA |  |
| KF026119 | N | ET | Gondar | 2009 | C-EA |  |
| KF026120 | N | ET | Gondar | 2009 | C-EA |  |
| KF026126 | N | ET | Gondar | 2009 | C-EA |  |
| KF026127 | N | ET | Gondar | 2009 | C-EA |  |
| KF026130 | N | ET | Gondar | 2009 | C-EA |  |
| KF026132 | N | ET | Gondar | 2009 | C-EA |  |
| KF026134 | N | ET | Gondar | 2009 | C-EA |  |
| KF026135 | D | ET | Gondar | 2009 | C-EA |  |
| KF026137 | N | ET | Gondar | 2009 | C-EA |  |
| KF026140 | N | ET | Gondar | 2009 | C-EA |  |
| KF026142 | N | ET | Gondar | 2009 | C-EA |  |
| KF026143 | N | ET | Gondar | 2009 | C-EA |  |
| KF026144 | N | ET | Gondar | 2009 | C-EA |  |
| KF026145 | N | ET | Gondar | 2009 | C-EA |  |
| KF026146 | N | ET | Gondar | 2009 | C-EA |  |
| KF026148 | N | ET | Gondar | 2009 | C-EA |  |
| KF026149 | N | ET | Gondar | 2009 | C-EA |  |
| KF026150 | N | ET | Gondar | 2009 | C-EA |  |
| KF026151 | N | ET | Gondar | 2009 | C-EA |  |
| KF026152 | N | ET | Gondar | 2009 | C-EA |  |
| KF026153 | N | ET | Gondar | 2009 | C-EA |  |
| KF026154 | N | ET | Gondar | 2009 | C-EA |  |
| KF026155 | N | ET | Gondar | 2009 | C-EA |  |
| KF026157 | N | ET | Gondar | 2009 | C-EA |  |
| KF026158 | N | ET | Gondar | 2009 | C-EA |  |
| KF026160 | N | ET | Gondar | 2009 | C-EA |  |
| KF026162 | N | ET | Gondar | 2009 | C-EA |  |
| KF026164 | N | ET | Gondar | 2009 | C-EA |  |
| KF026168 | D | ET | Gondar | 2009 | C-EA |  |
| KF026169 | N | ET | Gondar | 2009 | C-EA |  |
| KF026170 | N | ET | Gondar | 2009 | C-EA |  |
| KF026171 | N | ET | Gondar | 2009 | C-EA |  |
| KF026176 | N | ET | Gondar | 2009 | C-EA |  |
| KF026178 | N | ET | Gondar | 2009 | C-EA |  |
| KF026181 | N | ET | Gondar | 2009 | C-EA |  |
| KF026182 | N | ET | Gondar | 2009 | C-EA |  |
| KF026183 | N | ET | Gondar | 2009 | C-EA |  |
| KF026184 | D | ET | Gondar | 2009 | C-EA |  |
| KF026186 | N | ET | Gondar | 2009 | C-EA |  |
| KF026187 | N | ET | Gondar | 2009 | C-EA |  |
| KF026190 | N | ET | Gondar | 2009 | C-EA |  |
| KF026191 | N | ET | Gondar | 2009 | C-EA |  |
| KF026195 | N | ET | Gondar | 2009 | C-EA |  |
| KF026198 | N | ET | Gondar | 2009 | C-EA |  |
| KF026199 | N | ET | Gondar | 2009 | C-EA |  |
| KF241499 | N | NG |  | 2010 | C-EA |  |
| KF526219 | N | US |  | 2011 | C-EA |  |
| KF745350 | N | TH |  | 2013 | C-EA |  |
| KJ561132 | N | ETJM |  | 2012 | C-EA |  |
| KJ770424 | N | DE |  | 2007 | C-EA |  |
| KJ807652 | N | ET | Gondar | 2010 | C-EA |  |
| KJ807653 | D | ET | Gondar | 2010 | C-EA |  |
| KJ807655 | N | ET | Gondar | 2010 | C-EA |  |
| KJ807657 | D | ET | Gondar | 2010 | C-EA |  |
| KJ807659 | N | ET | Gondar | 2010 | C-EA |  |
| KJ807661 | N | ET | Gondar | 2010 | C-EA |  |
| KJ807662 | N | ET | Gondar | 2010 | C-EA |  |
| KJ807663 | N | ET | Gondar | 2010 | C-EA |  |
| KJ807664 | N | ET | Gondar | 2010 | C-EA |  |
| KJ807665 | N | ET | Gondar | 2010 | C-EA |  |
| KJ807668 | N | ET | Gondar | 2010 | C-EA |  |
| KJ807673 | N | ET | Gondar | 2010 | C-EA |  |
| KJ807675 | N | ET | Gondar | 2010 | C-EA |  |
| KJ807676 | N | ET | Metema | 2010 | C-EA |  |
| KJ807679 | N | ET | Metema | 2010 | C-EA |  |
| KJ807682 | N | ET | Metema | 2010 | C-EA |  |
| KJ807684 | N | ET | Metema | 2010 | C-EA |  |
| KJ807686 | N | ET | Metema | 2010 | C-EA |  |
| KJ807688 | D | ET | Metema | 2010 | C-EA |  |
| KJ807691 | N | ET | Gondar | 2010 | C-EA |  |
| KJ807704 | N | ET | Gondar | 2010 | C-EA |  |
| KJ807715 | N | ET | Gondar | 2010 | C-EA |  |
| KJ807743 | N | ET | Gondar | 2010 | C-EA |  |
| KP090065 | N | RU |  | 2014 | C-EA |  |
| KP411835 | N | SE |  | 2013 | C-EA |  |
| KT020933 | D | ET | Gondar | 2010 | C-EA |  |
| KT367544 | N | ET |  | 2013 | C-EA |  |
| KU498644 | D | GB |  | 2007 | C-EA |  |
| KU498708 | N | GB |  | 2007 | C-EA |  |
| KU498868 | N | GB |  | 2008 | C-EA |  |
| KU498984 | N | GB |  | 2008 | C-EA |  |
| U46016 | N | ET | Addis Ababa | 1986 | C-EA |  |
| AB285753 | N | ET | Gondar | 2003 | C'-ET |  |
| AB285758 | N | ET | Gondar | 2003 | C'-ET |  |
| AB285759 | N | ET | Gondar | 2003 | C'-ET |  |
| AB285760 | N | ET | Gondar | 2003 | C'-ET |  |
| AB285762 | N | ET | Gondar | 2003 | C'-ET |  |
| AB285763 | N | ET | Gondar | 2003 | C'-ET |  |
| AB285766 | N | ET | Gondar | 2003 | C'-ET |  |
| AB285767 | N | ET | Gondar | 2003 | C'-ET |  |
| AB285768 | N | ET | Gondar | 2003 | C'-ET |  |
| AB285771 | N | ET | Gondar | 2003 | C'-ET |  |
| AB285773 | N | ET | Gondar | 2003 | C'-ET |  |
| AB285780 | N | ET | Gondar | 2003 | C'-ET |  |
| AB285783 | N | ET | Gondar | 2003 | C'-ET |  |
| AB285786 | N | ET | Gondar | 2003 | C'-ET |  |
| AB285794 | N | ET | Gondar | 2003 | C'-ET |  |
| AB285796 | N | ET | Gondar | 2003 | C'-ET |  |
| AB285799 | N | ET | Gondar | 2003 | C'-ET |  |
| AB285800 | N | ET | Gondar | 2003 | C'-ET |  |
| AB285802 | N | ET | Gondar | 2003 | C'-ET |  |
| AB285803 | N | ET | Gondar | 2003 | C'-ET |  |
| AB285805 | N | ET | Gondar | 2003 | C'-ET |  |
| AB285817 | N | ET | Gondar | 2003 | C'-ET |  |
| AB285818 | N | ET | Gondar | 2003 | C'-ET |  |
| AB285819 | N | ET | Gondar | 2003 | C'-ET |  |
| AB285820 | N | ET | Gondar | 2003 | C'-ET |  |
| AB285823 | N | ET | Gondar | 2003 | C'-ET |  |
| AB285824 | N | ET | Gondar | 2003 | C'-ET |  |
| AB285825 | N | ET | Gondar | 2003 | C'-ET |  |
| AB285827 | N | ET | Gondar | 2003 | C'-ET |  |
| AB285829 | N | ET | Gondar | 2003 | C'-ET |  |
| AB285836 | N | ET | Gondar | 2003 | C'-ET |  |
| AB285838 | N | ET | Gondar | 2003 | C'-ET |  |
| AM285286 | N | DK |  | 2001 | C'-ET |  |
| AY102510 | N | SD |  | 1999 | C'-ET |  |
| AY165208 | N | SE |  | 1999 | C'-ET |  |
| AY165211 | N | SE |  | 1999 | C'-ET |  |
| AY242581* | N | ET | Jimma | 1996 | C'-ET |  |
| AY242582 | N | ET | Gondar | 1988 | C'-ET |  |
| AY242598* | N | ET | Arba Minch | 1996 | C'-ET |  |
| AY242604 | N | ET | Gondar | 1996 | C'-ET |  |
| AY255824 | N | IL |  | 1999 | C'-ET |  |
| AY749188 | N | BE |  | NA | C'-ET |  |
| DR010 | N | ET | Gondar | 2011 | C'-ET |  |
| DR018 | N | ET | Gondar | 2011 | C'-ET |  |
| DR030 | N | ET | Gondar | 2011 | C'-ET |  |
| DR048 | N | ET | Gondar | 2011 | C'-ET |  |
| DR053 | N | ET | Gondar | 2011 | C'-ET |  |
| DR060 | N | ET | Gondar | 2012 | C'-ET |  |
| DR062 | N | ET | Gondar | 2012 | C'-ET |  |
| DR069 | N | ET | Gondar | 2012 | C'-ET |  |
| DR071 | N | ET | Gondar | 2012 | C'-ET |  |
| DR075 | N | ET | Gondar | 2012 | C'-ET |  |
| DR077 | N | ET | Gondar | 2012 | C'-ET |  |
| DR083 | N | ET | Gondar | 2012 | C'-ET |  |
| DR091 | N | ET | Gondar | 2013 | C'-ET |  |
| DR518 | N | ET | Gondar | 2012 | C'-ET |  |
| DR520 | N | ET | Gondar | 2012 | C'-ET |  |
| DR526 | N | ET | Gondar | 2012 | C'-ET |  |
| EF369163 | N | CA |  | na | C'-ET |  |
| EU672623 | N | CZ |  | na | C'-ET |  |
| GQ398909 | N | DK |  | 2004 | C'-ET |  |
| GQ399666 | N | IT |  | 2005 | C'-ET |  |
| GQ400646 | N | SE |  | 2004 | C'-ET |  |
| GQ400909 | N | SE |  | 2003 | C'-ET |  |
| GQ462419 | N | GB |  | na | C'-ET |  |
| GU326127 | N | ES |  | 2008 | C'-ET |  |
| JQ698747 | N | SE |  | 2010 | C'-ET |  |
| JQ698796 | N | SE |  | 2007 | C'-ET |  |
| JX300863 | N | NO |  | 2006 | C'-ET |  |
| JX460000 | N | US |  | 2007 | C'-ET |  |
| JX460057 | N | US |  | 2008 | C'-ET |  |
| JX460166 | N | US |  | 2008 | C'-ET |  |
| JX460409 | N | US |  | 2010 | C'-ET |  |
| JX460662 | N | US |  | 2011 | C'-ET |  |
| JX460735 | N | US |  | 2011 | C'-ET |  |
| JX460743 | N | US |  | 2011 | C'-ET |  |
| KC340804 | N | ES |  | 2009 | C'-ET |  |
| KF026063 | N | ET | Gondar | 2009 | C'-ET |  |
| KF026067 | N | ET | Gondar | 2009 | C'-ET |  |
| KF026070 | N | ET | Gondar | 2009 | C'-ET |  |
| KF026071 | N | ET | Gondar | 2009 | C'-ET |  |
| KF026085 | N | ET | Gondar | 2009 | C'-ET |  |
| KF026089 | N | ET | Gondar | 2009 | C'-ET |  |
| KF026090 | N | ET | Gondar | 2009 | C'-ET |  |
| KF026091 | N | ET | Gondar | 2009 | C'-ET |  |
| KF026101 | N | ET | Gondar | 2009 | C'-ET |  |
| KF026105 | N | ET | Gondar | 2009 | C'-ET |  |
| KF026108 | N | ET | Gondar | 2009 | C'-ET |  |
| KF026112 | N | ET | Gondar | 2009 | C'-ET |  |
| KF026117 | N | ET | Gondar | 2009 | C'-ET |  |
| KF026125 | N | ET | Gondar | 2009 | C'-ET |  |
| KF026129 | N | ET | Gondar | 2009 | C'-ET |  |
| KF026131 | N | ET | Gondar | 2009 | C'-ET |  |
| KF026133 | N | ET | Gondar | 2009 | C'-ET |  |
| KF026138 | N | ET | Gondar | 2009 | C'-ET |  |
| KF026139 | N | ET | Gondar | 2009 | C'-ET |  |
| KF026141 | N | ET | Gondar | 2009 | C'-ET |  |
| KF026147 | N | ET | Gondar | 2009 | C'-ET |  |
| KF026156 | N | ET | Gondar | 2009 | C'-ET |  |
| KF026159 | N | ET | Gondar | 2009 | C'-ET |  |
| KF026161 | N | ET | Gondar | 2009 | C'-ET |  |
| KF026165 | N | ET | Gondar | 2009 | C'-ET |  |
| KF026166 | D | ET | Gondar | 2009 | C'-ET |  |
| KF026172 | N | ET | Gondar | 2009 | C'-ET |  |
| KF026173 | N | ET | Gondar | 2009 | C'-ET |  |
| KF026174 | N | ET | Gondar | 2009 | C'-ET |  |
| KF026175 | N | ET | Gondar | 2009 | C'-ET |  |
| KF026177 | N | ET | Gondar | 2009 | C'-ET |  |
| KF026179 | N | ET | Gondar | 2009 | C'-ET |  |
| KF026188 | N | ET | Gondar | 2009 | C'-ET |  |
| KF026192 | N | ET | Gondar | 2009 | C'-ET |  |
| KF026197 | N | ET | Gondar | 2009 | C'-ET |  |
| KF026204 | N | ET | Gondar | 2009 | C'-ET |  |
| KJ561130 | N | ET | Jimma | 2012 | C'-ET |  |
| KJ561135 | D | ET | Jimma | 2011 | C'-ET |  |
| KJ723062 | N | US |  | 2010 | C'-ET |  |
| KJ807649 | N | ET | Gondar | 2010 | C'-ET |  |
| KJ807651 | N | ET | Gondar | 2010 | C'-ET |  |
| KJ807656 | N | ET | Gondar | 2010 | C'-ET |  |
| KJ807658 | N | ET | Gondar | 2010 | C'-ET |  |
| KJ807669 | N | ET | Gondar | 2010 | C'-ET |  |
| KJ807671 | N | ET | Gondar | 2010 | C'-ET |  |
| KJ807683 | N | ET | Metema | 2010 | C'-ET |  |
| KJ807689 | N | ET | Metema | 2010 | C'-ET |  |
| KJ807695 | N | ET | Gondar | 2010 | C'-ET |  |
| KJ807703 | N | ET | Gondar | 2010 | C'-ET |  |
| KJ807706 | N | ET | Gondar | 2010 | C'-ET |  |
| KJ807719 | N | ET | Metema | 2010 | C'-ET |  |
| KJ807722 | N | ET | Metema | 2010 | C'-ET |  |
| KJ807737 | D | ET | Gondar | 2010 | C'-ET |  |
| KJ807761 | N | ET | Metema | 2010 | C'-ET |  |
| KP411831 | N | SE |  | 2007 | C'-ET |  |
| KT020931 | N | ET | Gondar | 2010 | C'-ET |  |
| KU498572 | N | GB |  | 2008 | C'-ET |  |
| KU498673 | N | GB |  | 2008 | C'-ET |  |
| KU498692 | N | GB |  | 2008 | C'-ET |  |
| KU498709 | N | GB |  | 2008 | C'-ET |  |
| KU498943 | N | GB |  | 2008 | C'-ET |  |
| KU499153 | N | GB |  | 2008 | C'-ET |  |
| AB285752 | N | ET | Gondar | 2003 | C-SA |  |
| AB285797 | N | ET | Gondar | 2003 | C-SA |  |
| AB747502 | N | PH |  | 2011 | C-SA |  |
| AF411967 | N | ZA |  | 1999 | C-SA |  |
| AJ419450 | N | DK |  | 2000 | C-SA |  |
| AM260232 | N | BI |  | 2002 | C-SA |  |
| AY165255 | N | SE |  | 2001 | C-SA |  |
| AY196509 | N | ZA |  | 2001 | C-SA |  |
| AY253317 | N | TZ |  | 2001 | C-SA |  |
| AY713415 | N | SO |  | 1989 | C-SA |  |
| AY713416 | N | SN |  | 1990 | C-SA |  |
| AY746373* | N | IN |  | 2001 | C-SA |  |
| AY749195 | N | BE |  | na | C-SA |  |
| AY756836 | N | MW |  | 2000 | C-SA |  |
| AY958383 | N | MW |  | na | C-SA |  |
| AY967806 | N | CN |  | 1998 | C-SA |  |
| DQ351226* | N | ZA |  | 2003 | C-SA |  |
| DR001 | N | ET | Gondar | 2011 | C-SA |  |
| DR016 | N | ET | Gondar | 2011 | C-SA |  |
| DR039 | N | ET | Gondar | 2011 | C-SA |  |
| DR043 | N | ET | Gondar | 2011 | C-SA |  |
| DR049 | N | ET | Gondar | 2011 | C-SA |  |
| DR058 | D | ET | Gondar | 2012 | C-SA |  |
| DR070 | N | ET | Gondar | 2012 | C-SA |  |
| DR081 | N | ET | Gondar | 2012 | C-SA |  |
| DR085 | N | ET | Gondar | 2012 | C-SA |  |
| DR087 | N | ET | Gondar | 2012 | C-SA |  |
| DR092 | N | ET | Gondar | 2013 | C-SA |  |
| DR516 | N | ET | Gondar | 2011 | C-SA |  |
| DR522 | N | ET | Gondar | 2012 | C-SA |  |
| EF186956 | N | IN |  | 2004 | C-SA |  |
| EF602190 | N | ZA |  | 2002 | C-SA |  |
| EF602611 | N | MW |  | 2002 | C-SA |  |
| EU683757 | N | IN |  | 2007 | C-SA |  |
| FJ199537 | N | ZA |  | 2002 | C-SA |  |
| FJ199625 | N | ZA |  | 2003 | C-SA |  |
| GQ433802 | N | ZM |  | 2005 | C-SA |  |
| GQ433812 | N | ZM |  | 2005 | C-SA |  |
| GQ433835 | N | ZM |  | 2005 | C-SA |  |
| GQ433857 | N | ZM |  | 2005 | C-SA |  |
| GQ462158 | N | GB |  | na | C-SA |  |
| HE588162 | N | SN |  | na | C-SA |  |
| HG780683 | N | BI |  | na | C-SA |  |
| HM119735 | N | ZM |  | 2008 | C-SA |  |
| HM119750 | N | ZM |  | 2008 | C-SA |  |
| HM119763 | N | ZM |  | 2008 | C-SA |  |
| HM119826 | N | ZM |  | 2008 | C-SA |  |
| HM119855 | N | ZM |  | 2008 | C-SA |  |
| HM119933 | N | ZM |  | 2008 | C-SA |  |
| HM119994 | N | ZM |  | 2008 | C-SA |  |
| HM120014 | N | ZM |  | 2008 | C-SA |  |
| HM120065 | N | ZM |  | 2008 | C-SA |  |
| HM120097 | N | ZM |  | 2008 | C-SA |  |
| HM572402 | N | TZ |  | 2009 | C-SA |  |
| HQ700060 | N | PT |  | na | C-SA |  |
| JF960556 | N | ZA |  | 2008 | C-SA |  |
| JN023035 | N | NP |  | 2010 | C-SA |  |
| JN247071 | N | MW |  | 2009 | C-SA |  |
| JN314975 | N | IN |  | 2008 | C-SA |  |
| JN408426 | N | IN |  | 2007 | C-SA |  |
| JN408434 | N | IN |  | 2007 | C-SA |  |
| JQ698790 | N | SE |  | 2010 | C-SA |  |
| JQ698791 | N | SE |  | 2007 | C-SA |  |
| JX460669 | D | US |  | 2011 | C-SA |  |
| KC424147 | N | ZA |  | 2008 | C-SA |  |
| KC517051 | N | KE |  | 2006 | C-SA |  |
| KF026081 | N | ET | Gondar | 2009 | C-SA |  |
| KF026086 | N | ET | Gondar | 2009 | C-SA |  |
| KF026087 | N | ET | Gondar | 2009 | C-SA |  |
| KF026094 | N | ET | Gondar | 2009 | C-SA |  |
| KF026095 | N | ET | Gondar | 2009 | C-SA |  |
| KF026099 | N | ET | Gondar | 2009 | C-SA |  |
| KF026118 | N | ET | Gondar | 2009 | C-SA |  |
| KF026122 | N | ET | Gondar | 2009 | C-SA |  |
| KF026163 | N | ET | Gondar | 2009 | C-SA |  |
| KF026167 | N | ET | Gondar | 2009 | C-SA |  |
| KF531054 | N | TZ |  | 2005 | C-SA |  |
| KF736543 | N | ZA |  | 2006 | C-SA |  |
| KF736547 | N | ZA |  | 2006 | C-SA |  |
| KF927291 | N | MZ |  | 2009 | C-SA |  |
| KF927314 | N | MZ |  | 2009 | C-SA |  |
| KJ807667 | D | ET | Gondar | 2010 | C-SA |  |
| KJ807672 | N | ET | Gondar | 2010 | C-SA |  |
| KJ807681 | N | ET | Metema | 2010 | C-SA |  |
| KM049902 | N | ZM |  | 2005 | C-SA |  |
| KM049903 | N | ZM |  | 2005 | C-SA |  |
| KM050082 | N | ZM |  | 2005 | C-SA |  |
| KM050125 | N | ZM |  | 2006 | C-SA |  |
| KM050372 | N | ZM |  | 2003 | C-SA |  |
| KM050388 | N | ZM |  | 2002 | C-SA |  |
| KM050398 | N | ZM |  | 2005 | C-SA |  |
| KM050474 | N | ZM |  | 2003 | C-SA |  |
| KM050516 | N | ZM |  | 2004 | C-SA |  |
| KM050645 | N | ZM |  | 2000 | C-SA |  |
| KM050719 | N | ZM |  | 2005 | C-SA |  |
| KM050739 | N | ZM |  | 2006 | C-SA |  |
| KP109480 | N | IN |  | 2000 | C-SA |  |
| KP227625 | N | IN |  | na | C-SA |  |
| KR860717 | N | BW |  | 2014 | C-SA |  |
| KR860725 | N | BW |  | 2014 | C-SA |  |
| KR861000 | N | BW |  | 2008 | C-SA |  |
| KR861056 | N | BW |  | 2008 | C-SA |  |
| KR861180 | N | BW |  | 2008 | C-SA |  |
| KR861217 | N | BW |  | 2011 | C-SA |  |
| KT365509 | N | MZ |  | 2008 | C-SA |  |
| KU498864 | N | GB |  | 2008 | C-SA |  |
| KU498882 | N | GB |  | 2009 | C-SA |  |
| KU498891 | N | GB |  | 2007 | C-SA |  |
| AB285765 | nd | ET | Gondar | 2003 | PR-CC |  |
| AB285790 | nd | ET | Gondar | 2003 | PR-CC |  |
| AB285793 | nd | ET | Gondar | 2003 | PR-CC |  |
| AB285812 | nd | ET | Gondar | 2003 | PR-CC |  |
| AB285813 | nd | ET | Gondar | 2003 | PR-CC |  |
| DR044 | N | ET | Gondar | 2011 | PR-CC |  |
| DR050 | N | ET | Gondar | 2011 | PR-CC |  |
| DR051 | N | ET | Gondar | 2011 | PR-CC |  |
| DR528 | N | ET | Gondar | 2012 | PR-CC |  |
| KF026066 | nd | ET | Gondar | 2009 | PR-CC |  |
| KF026078 | nd | ET | Gondar | 2009 | PR-CC |  |
| KF026084 | nd | ET | Gondar | 2009 | PR-CC |  |
| KF026086 | nd | ET | Gondar | 2009 | PR-CC |  |
| KF026088 | nd | ET | Gondar | 2009 | PR-CC |  |
| KF026094 | nd | ET | Gondar | 2009 | PR-CC |  |
| KF026100 | nd | ET | Gondar | 2009 | PR-CC |  |
| KF026121 | nd | ET | Gondar | 2009 | PR-CC |  |
| KF026128 | nd | ET | Gondar | 2009 | PR-CC |  |
| KF026136 | nd | ET | Gondar | 2009 | PR-CC |  |
| KF026180 | nd | ET | Gondar | 2009 | PR-CC |  |
| KF026185 | nd | ET | Gondar | 2009 | PR-CC |  |
| KF026193 | nd | ET | Gondar | 2009 | PR-CC |  |
| KF026194 | nd | ET | Gondar | 2009 | PR-CC |  |
| KF026196 | nd | ET | Gondar | 2009 | PR-CC |  |
| KJ807732 | nd | ET | Gondar | 2010 | PR-CC |  |
| KJ807733 | nd | ET | Gondar | 2010 | PR-CC |  |
| KJ807734 | nd | ET | Gondar | 2010 | PR-CC |  |
| KJ807735 | nd | ET | Gondar | 2010 | PR-CC |  |
| KJ807736 | nd | ET | Gondar | 2010 | PR-CC |  |
| KJ807738 | nd | ET | Gondar | 2010 | PR-CC |  |
| KJ807739 | nd | ET | Gondar | 2010 | PR-CC |  |
| KJ807740 | nd | ET | Gondar | 2010 | PR-CC |  |
| KJ807741 | nd | ET | Gondar | 2010 | PR-CC |  |
| KJ807742 | nd | ET | Gondar | 2010 | PR-CC |  |
| KJ807744 | nd | ET | Gondar | 2010 | PR-CC |  |
| KJ807745 | nd | ET | Gondar | 2010 | PR-CC |  |
| KJ807746 | nd | ET | Gondar | 2010 | PR-CC |  |
| KJ807747 | nd | ET | Gondar | 2010 | PR-CC |  |
| KJ807748 | nd | ET | Gondar | 2010 | PR-CC |  |
| KJ807749 | nd | ET | Gondar | 2010 | PR-CC |  |
| KJ807750 | nd | ET | Gondar | 2010 | PR-CC |  |
| KJ807751 | nd | ET | Gondar | 2010 | PR-CC |  |
| KJ807752 | nd | ET | Gondar | 2010 | PR-CC |  |
| KJ807753 | nd | ET | Gondar | 2010 | PR-CC |  |
| KJ807754 | nd | ET | Gondar | 2010 | PR-CC |  |
| KJ807755 | nd | ET | Gondar | 2010 | PR-CC |  |
| KJ807756 | nd | ET | Gondar | 2010 | PR-CC |  |
| KJ807757 | nd | ET | Gondar | 2010 | PR-CC |  |
| KJ807758 | nd | ET | Gondar | 2010 | PR-CC |  |
| KJ807759 | nd | ET | Gondar | 2010 | PR-CC |  |
| KJ807760 | nd | ET | Gondar | 2010 | PR-CC |  |
| KJ807762 | nd | ET | Gondar | 2010 | PR-CC |  |
| KJ807763 | nd | ET | Gondar | 2010 | PR-CC |  |
| KJ807764 | nd | ET | Gondar | 2010 | PR-CC |  |
| KJ807765 | nd | ET | Gondar | 2010 | PR-CC |  |
| KJ807766 | nd | ET | Gondar | 2010 | PR-CC |  |
| KJ807767 | nd | ET | Gondar | 2010 | PR-CC |  |
| KJ807768 | nd | ET | Gondar | 2010 | PR-CC |  |
| KJ807769 | nd | ET | Gondar | 2010 | PR-CC |  |
| KJ807770 | nd | ET | Gondar | 2010 | PR-CC |  |
| KJ807771 | nd | ET | Gondar | 2010 | PR-CC |  |
| KJ807772 | nd | ET | Gondar | 2010 | PR-CC |  |
| DR059 | N | ET | Gondar | 2012 | A/C |  |
| DR061 | N | ET | Gondar | 2012 | A/C |  |
| DR064 | N | ET | Gondar | 2012 | A-like |  |
| DR055 | D | ET | Gondar | 2012 | B-like |  |
| Taxa: GenBank Accession Number or study code (present study) | | | | | | |
| DR (Drug resistance): N, No; D, DRM | | | | | | |
| Country: two-letter country code (iso 3166-2); https://datahub.io/core/country-list | | | | | | |
| City: If ET sequence, the city where the sample was collected | | | | | | |
| Collection Year: Year of sample collection | | | | | | |
| Subtype/Clade: results of phylogenetic subtyping; C-EA: East African subtype C sequence; C'-ET: Ethiopian subtype C clade; C-SA: Southern African subtype C clade; PR-CC: putatvie subtypeC-subtypeC recombinant | | | | | | |
