# Supplementary material for: HIV-genetic diversity and drug resistance transmission clusters in Gondar, Northern Ethiopia, 2003-2013
Source: PLoS One. 2018 Oct 10;13(10):e0205446. doi: 10.1371/journal.pone.0205446 (PMC6179264; doi:10.1371/journal.pone.0205446)
Supplement: S3 Table — (DOCX) [file pone.0205446.s004.docx]

| **S3 Table. Taxa associated with clusters** | | | |
| --- | --- | --- | --- |
| **Cluster^1^** | **Taxa^2^** | **N** | **DRM^3^** |
| C-EA-1 | KF026113,KF026115,KF026143 | 3 |  |
| C-EA-2 | KF026184 (DR), AB285787, AB285788 | 3 | F53L |
| C-EA-2a | AB285787,AB285788 | 2 |  |
| C-EA-3 | KF026182,KF026183 | 2 |  |
| C-EA-4 | AB285774,AB285831 | 2 |  |
| C-EA-5 | KF026082,KF026083 | 2 |  |
| C-EA-6 | DR507, DR510, KF026061, KJ807743 | 4 |  |
| C-EA-7 | AB285811,KF026135 (DR) | 2 | D67E (1) |
| C-EA-8 | KF026109,KF026110 | 2 |  |
| C-EA-9 | AB285843,KF026076 | 2 |  |
| C-EA-10 | AB285778,KF026111 | 2 |  |
| C-EA-11 | AB285795,KF026137 | 2 |  |
| C-EA-12 | AB285755, AB285761, AB285806, AB285807, AB285822, AY371691, DR066, KF026116, KF026140, KF026142, KF026157, KF026187, KJ807652, KJ807659, KJ807673, KJ807676, KT367544, AB285770, AB285798 (DR), DR513, KF026077, KF026098 (DR), KF026144, KF026168 (DR), KF026171, KJ807688 (DR), AB285775, AB285776, AB285837, KF026154, DR088 (DR), KF026153, AB285837, KF026154, DR088, KF026153, KF026079, KF026130 | 32 | G190A (4), Y181C (1) |
| C-EA-12a | AB285770, AB285798 (DR), DR513, KF026077, KF026098 (DR), KF026144, KF026168 (DR), KF026171, KJ807688 (DR) | 9 | G190A (4) |
| C-EA-12b | AB285837, KF026154 | 2 |  |
| C-EA-12c | DR088 (DR), KF026153 | 2 | Y181C (1) |
| C-EA-12d | KF026079, KF026130 | 2 |  |
| C-EA-13 | AB285781, AB285821, DR052, KF026074, KJ807665, KJ807691, KF026079, KF026130 | 8 |  |
| C-EA-13a | KF026079, KF026130 | 2 |  |
| C-EA-14 | KF026075,KF026104 | 2 |  |
| C-EA-15 | AB285769,AB285815, KF026092,KF026093 | 2 |  |
| C-EA-15a | KF026092,KF026093 | 2 |  |
| C-SA-1 | AB285752, AB285797, AY165255, DR049, DR058 (DR), DR070 ,DR081 ,DR522, JQ698790, KF026087, KF026163, KF026167, KJ807667 (DR) | 13 | K103N (1), G190E (1) |
| C-SA-2 | DR043,DR085 | 2 |  |
| C'-ET-1 | KF026090,KF026091 | 2 |  |
| C'-ET-2 | DR060,KJ807703 | 2 |  |
| C'-ET-3 | KF026156,KT020931 | 2 |  |
| C'-ET-4 | KF026131,KF026161 | 2 |  |
| C'-ET-5 | KF026063,KF026125 | 2 |  |
| C'-ET-6 | AB285818,KF026177 | 2 |  |
| C'-ET-7 | AB285767,KJ807671 | 2 |  |
| C'-ET-8 | DR091,KJ807695 | 2 |  |
| C'-ET-9 | DR030,KJ807656 | 2 |  |
| C'-ET-10 | KF026085,KF026101 | 2 |  |
| C'-ET-11 | AB285803,KF026166 (DR) | 2 | L210W+M46I (1) |

^1^Cluster refers to the subtype C clade (C-EA, C-SA or C’-ET) followed by cluster number as shown in Fig. 2.
^2^ Taxa refers to Genbank Accession Numbers or study codes (current study).
^3^ DRM, Drug resistance mutations. Mutations were defined by the Stanford Genotypic Resistance Interpretation Algorithm (http://hivdb.stanford.edu/pages/algs/HIVdb.html) using the calibrated population resistance (CPR) tool version 6.0 (http://cpr.stanford.edu/cpr/servlet/CPR), based on the WHO surveillance transmitted drug resistance mutation list of 2009.
