# Supplementary material for: HIV-genetic diversity and drug resistance transmission clusters in Gondar, Northern Ethiopia, 2003-2013
Source: PLoS One. 2018 Oct 10;13(10):e0205446. doi: 10.1371/journal.pone.0205446 (PMC6179264; doi:10.1371/journal.pone.0205446)
Supplement: S1 Fig — (DOCX) [file pone.0205446.s005.docx]

**S1 Fig.**


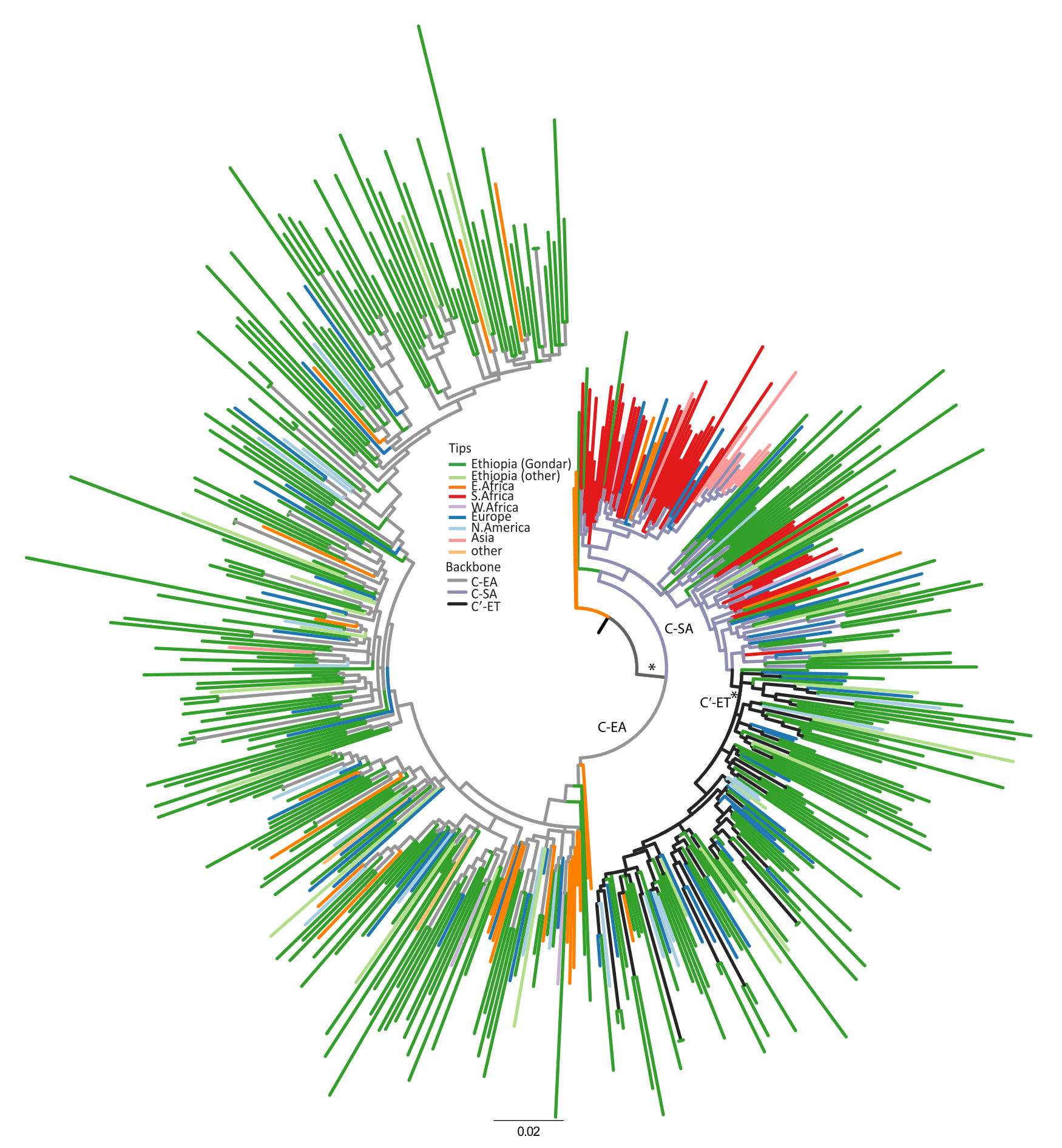


**S1 Fig. Phylogenetic subtyping of sequences obtained from Gondar 2003-2013**. The data set represented non-recombinant subtype C sequences of the current study (n=59), three previous studies from Gondar 2003-2010 (n=240) and a Genbank reference data set (n=192), resulting in a final data set of 491 sequences collected 1986-2013 (n=301 from Gondar; S1 Table). The maximum likelihood tree using all 491 sequences revealed that sequences fell into one of the three subtype C clades. Branches defining the t major clades are indicated with an asterisk, corresponding to a branch support (aLRT-SH) >0.9. Tips are coloured according to collection place, while the backbone of each clade is indicated in different shades of grey. The colour codes are indicated at the centre of the tree. The scale bar represents 0.02 substitutions/site.
