# Supplementary material for: HIV-genetic diversity and drug resistance transmission clusters in Gondar, Northern Ethiopia, 2003-2013
Source: PLoS One. 2018 Oct 10;13(10):e0205446. doi: 10.1371/journal.pone.0205446 (PMC6179264; doi:10.1371/journal.pone.0205446)
Supplement: S2 Fig — (DOCX) [file pone.0205446.s006.docx]

**S2 Fig**


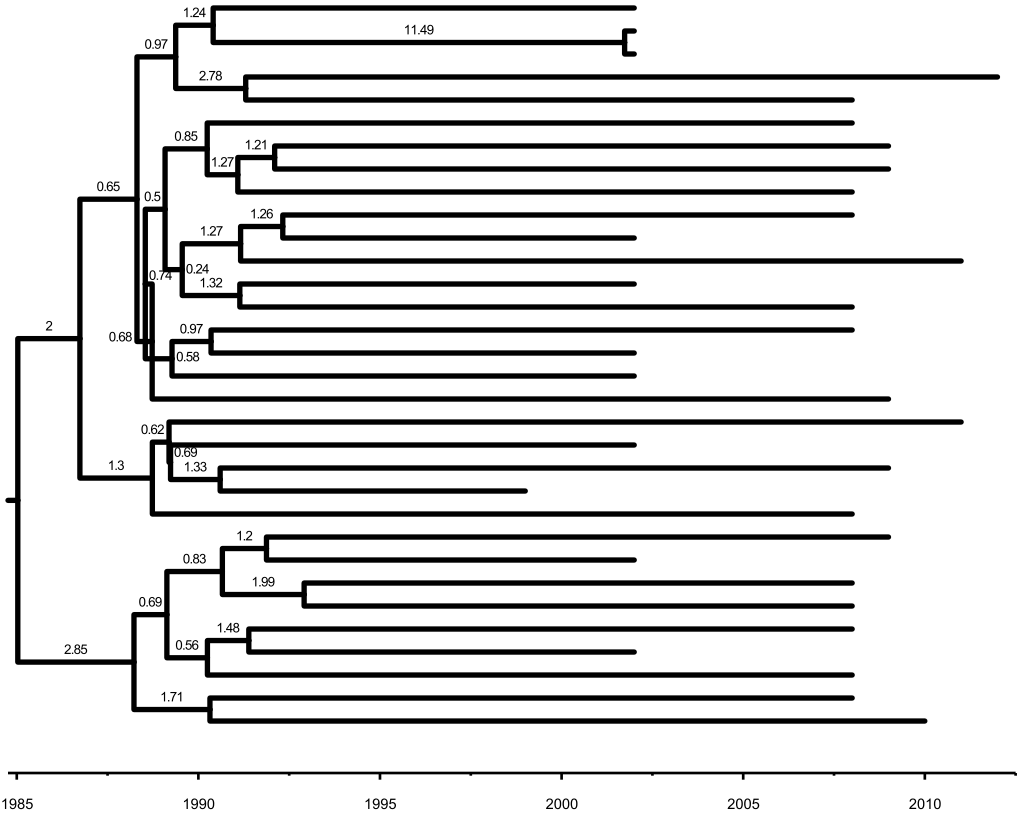


**S6 Fig. Maximum clade credibility tree of C-EA Cluster 12.** Internode intervals (in years) are indicated above each branch. The time scale below the tree is shown in calendar years.
